# Thirtieth Anniversary of the Discovery of Laxaphycins. Intriguing Peptides Keeping a Part of Their Mystery

**DOI:** 10.3390/md19090473

**Published:** 2021-08-24

**Authors:** Laurine Darcel, Sanjit Das, Isabelle Bonnard, Bernard Banaigs, Nicolas Inguimbert

**Affiliations:** CRIOBE, USR EPHE-UPVD-CNRS 3278, Université de Perpignan Via Domitia, 58 Avenue Paul Alduy, 66860 Perpignan, France; sanjitpharma002@gmail.com (S.D.); isabelle.bonnard@univ-perp.fr (I.B.); banaigs@univ-perp.fr (B.B.)

**Keywords:** laxaphycin, cyanobacteria, synthesis, biosynthesis, marine natural product, peptide, non ribosomal peptide synthase, biological activity, D-peptidase

## Abstract

Lipopeptides are a class of compounds generally produced by microorganisms through hybrid biosynthetic pathways involving non-ribosomal peptide synthase and a polyketyl synthase. Cyanobacterial-produced laxaphycins are examples of this family of compounds that have expanded over the past three decades. These compounds benefit from technological advances helping in their synthesis and characterization, as well as in deciphering their biosynthesis. The present article attempts to summarize most of the articles that have been published on laxaphycins. The current knowledge on the ecological role of these complex sets of compounds will also be examined.

## 1. Introduction

The 1970s are considered as the corner stone of the research devoted to secondary metabolites extracted from the marine environment. Since this date, it has been demonstrated that marine secondary metabolites have a high biological activity and a chemodiversity unequalled at the terrestrial level. These marine natural products can be used in applications such as drugs in the fight against cancer, virus, or bacterial infections but also as cosmetics [1,2,3,4]. Cyanobacteria that represent at least 50% of the aquatic biomass are effective producers of secondary metabolites that can be used as therapeutic agents [5,6,7,8], among which peptides isolated from various strains all over the world [9]. These peptides are often synthesized by non-ribosomal peptide synthetase (NRPS) or mixed biosynthetic pathways that involve polyketide synthase (PKS) operating in conjunction with NRPS [10,11,12]. These particular biosyntheses allow the assembly of complex peptides containing proteinogenic and non-proteinogenic amino acids such as α,β-dehydro-, *N*-methylated, and β-hydroxylated amino acids. The presence of an amino acid bearing a fatty sidechain within the sequence qualified them as lipopeptides. Furthermore, combining these specific amino acids with a cyclic structure confers them a restricted conformation that supports their biological activity and reduces the risk of enzymatic degradation, which makes these peptides very attractive for pharmacological research [13,14,15]. In addition, β-amino fatty acids induce lipophilicity, which leads to interactions with cell membranes and counterbalance the hydrophilic character of peptides [16]. However, the mechanism of action of cyclic lipopeptides is often not well known. It is therefore necessary to understand the mechanisms of action and to determine potential biological targets. In order to study lipopeptides more extensively, researchers need to have sufficient quantities of them, which is often difficult in the case of natural molecules, either because of their low concentration in cyanobacterial extracts, or because of the difficulty in harvesting these organisms at specific locations and seasons. It is therefore important to develop efficient synthesis routes for these compounds or to express them in host organisms.

Laxaphycins (lax) and their derivatives constitute one example of lipopeptides. The purpose of this review is to summarize the thirty-year history of laxaphycins and their derivatives, from their discovery and characterization to the determination of their different biological activities, including the description of their chemical synthesis and their biosynthesis.

## 2. Discovery and Characterization of Laxaphycins and Their Derivatives

Most of the laxaphycin derivative structures have been determined thanks to a combination of approaches based on multidimensional ^1^H and ^13^C NMR and high resolution mass spectrometry [17]. Peptide hydrolysis and amino acid derivatization by the advanced Marfey method allowed the determination of amino acid stereochemistry [18].

### 2.1. Laxaphycins

#### 2.1.1. Cyclic Laxaphycins

Laxaphycins were first isolated by Moore’s Hawaiian group in 1992 [19,20]. They were extracted from the cyanobacterium *Anabaena laxa*, collected in mud on the campus of the University of Hawaii. After successive fractionation steps, four major laxaphycins A, B, D, and E were isolated from about thirteen other similar peptides. The raw cyclic structures of the four laxaphycins were determined. These four peptides could already be separated into two subfamilies, laxaphycins with 11 residues (lax A, E) and laxaphycins containing 12 residues (lax B, D), each of them containing in their sequences at least 50% of non-ribosomal amino acids. Laxaphycin A is composed of a 3-aminooctanoic acid residue (Aoc), two homoserine (Hse), a *E*-dehydrobutyrine residue (Dhb), a 4-hydroxyproline (Hyp), and six more standard amino acids with the following sequence (Aoc-Hse-*E*-Dhb-Hyp-Hse-Phe-Leu-Ile-Ile-Leu-Gly). The two isoleucines are diastereoisomers. Laxaphycin E showed a unique difference with laxaphycin A, the substitution of the 3-aminooctanoic acid (Aoc) by a 3-aminodecanoic acid (Ade). Laxaphycin B contains the non-proteinogenic amino acids 3-aminodecanoic acid (Ade), 3-hydroxyleucine (Hle), 3-hydroxyasparagine (Hasn), and *N*-methylisoleucine ((*N*)-MeIle) as well as six natural amino acids, with the sequence (Ade-Val-Hle-Gln-(*N*)-MeIle-Hasn-Thr-Pro-Leu-Thr). Laxaphycin D differentiated from laxaphycin B by a 3-aminooctanoic acid that replaces the 3-aminodecanoic acid.

**Figure 1:**
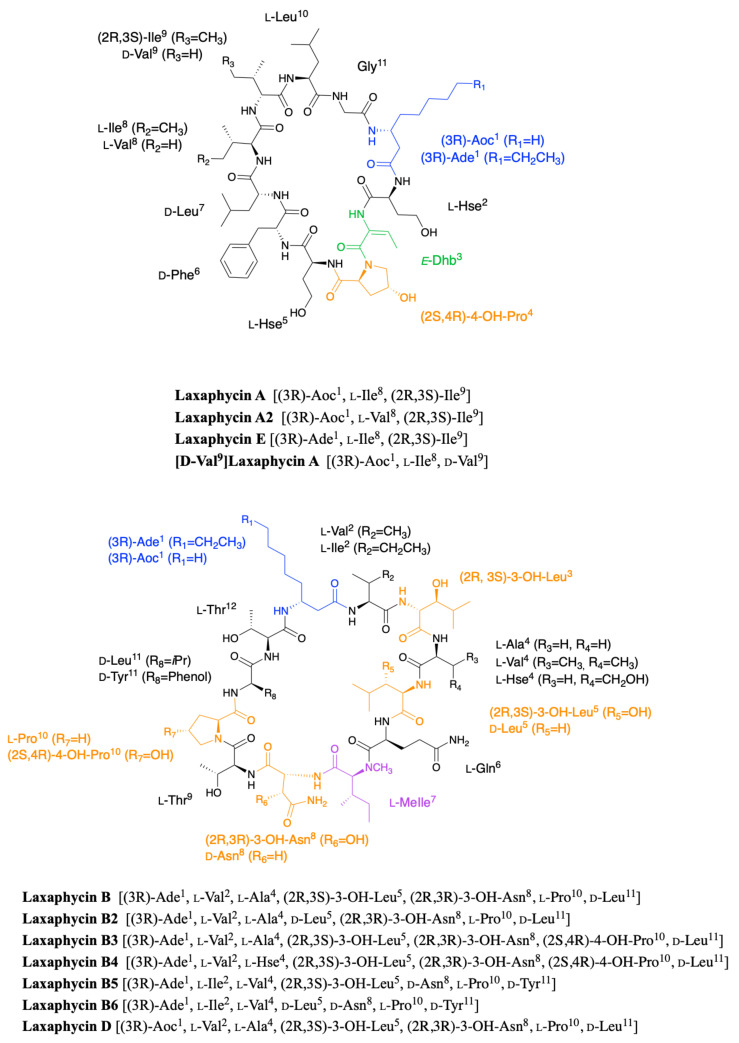
Structure of cyclic laxaphycins A and B.

It is only in 1997 that Bonnard et al. identified the complete structure and assigned the absolute stereochemistry of the amino acids constituting the laxaphycins A and B sequence [21]. The two peptides were extracted from an assemblage of the marine cyanobacteria *Lyngbya majuscula (L. majuscula)* and *Anabaena torulosa (A. torulosa)*, collected in Moorea (French Polynesia), but repeated harvesting at Moorea island highlighted *A. torulosa* as the only producer of laxaphycins. Different experiments including mass spectrometry and NMR confirmed the established gross structure, whereas combining the previous results with Marfey’s analysis gave access to the stereochemistry of all amino acids. Thus, for laxaphycin A, 3-aminooctanoic acid, phenylalanine, and leucine in positions 1, 6, and 7, respectively were shown to be of R-configuration. Isoleucine in position 8 is of (2S,3S)-configuration while isoleucine in position 9 has a (2R,3S) configuration, the other amino acids of the sequence having a classical S-configuration. It is worth noting that for laxaphycin A, hydrophobic residues are located on the same side of the peptide. In laxaphycin B the stereochemistry of 3-hydroxyleucine in position 3 was (2S,3S) while 3-hydroxyleucine in position 5 was (2R,3S). The 3-hydroxyasparagine was stereochemically (2R,3R). An alternation of L and D stereochemistry can be seen along the peptide sequence, with (3R)-Ade^1^, (2R,3S)-3-OH-Leu^5^, (2R,3R)-3-OH-Asn^8^ and d-Leu^11^, and the hydrophobic/hydrophilic character of amino acids is also alternated within the sequence. A further study of the biological activities of laxaphycins produced by cyanobacteria was carried out by the same group in 2007, giving rise at the same time to two new laxaphycins named laxaphycins B2 and B3 (Figure 1) [22]. Laxaphycin B2 differs from laxaphycin B only by the replacement of (2R,3S)-3-hydroxyleucine to d-leucine in position 5. Laxaphycin B3 contains a (2S,4R)-4-hydroxyproline in position 10 instead of the proline found in laxaphycin B. In these two new laxaphycins, the 3-hydroxyleucine in position 3 is of the (2R,3S) configuration whereas the one described in laxaphycin B is of the (2S,3S) configuration. 

In order to further study the ecological role of laxaphycin B and its potential in the discovery of new anti-cancer agents, a total synthesis was achieved in 2013 highlighting a structural misassignment for the absolute configuration of one amino acid [23,24]. Indeed, the comparison of synthetic and natural laxaphycin B showed that laxaphycin B had a (2R,3S)-3-hydroxyleucine in position 3 whereas it had been described with a (2S,3S) configuration (Figure 1).

About a decade later, new analogues of the laxaphycin family were reported, having little difference with the already known laxaphycins [25,26]. Laxaphycins A2 and B4 were discovered in an extract of the cyanobacterium *Hormothamnion enteromorphoides* collected in the Gulf of Mexico. Laxaphycin A2 contains an l-valine in position 8 while laxaphycin A contains an l-isoleucine (Figure 1) [25]. Laxaphycin B4 is analogous to laxaphycin B3, with a homoserine in position 4 replacing the alanine (Figure 1) [25]. Two new peptides laxaphycins B5 and B6 isolated from a freshwater cyanobacterium, *Phormidium* sp., UIC 10484 completed this series [26]. Laxaphycin B5 is derived from laxaphycin B and contains isoleucine instead of valine in position 2, valine in position 4 instead of alanine, asparagine instead of 3-hydroxyasparagine in position 8, and tyrosine in position 11 instead of leucine. Similarly, laxaphycin B6 combines the same four previous modifications observed in laxaphycin B5 and a d-leucine in position 5 that made it one of the more distant compound from laxaphycin B. It is worth to note that all the observed modifications are made with maintenance of stereochemistry and that the amino acids are often isosteric. The tyrosine and leucine in position 11 are both d-configuration, as are asparagine and 3-hydroxyasparagine in position 8.

One year later, an extract of *A. torulosa* collected in Moorea (French Polynesia) revealed two cyclic laxaphycins-A type peptides, one of which was the previously described laxaphycin A2 [27]. The other, named [d-Val^9^]laxaphycin A, showed a very similar structure, but NMR and MSMS analyses suggested the presence of d-valine in position 9 instead of (2R,3S)-isoleucine, while keeping (2S,3S)-isoleucine in position 8 (Figure 1). These two new cyclic laxaphycin A were not found in other samples of *Anabaena* sp. collected at different locations in the lagoon of Moorea [28]. The production of these peptides cannot yet be explained. Peptides with close structures have been found in other cyanobacteria such as *Hormothamnion enteromorphoides* [29] or *Lyngbya confervoides* [30]. A horizontal gene transfer between cyanobacteria could be an explanation for the presence of these peptides within different cyanobacteria strains [31].

#### 2.1.2. Acyclic Laxaphycins

**Figure 2:**
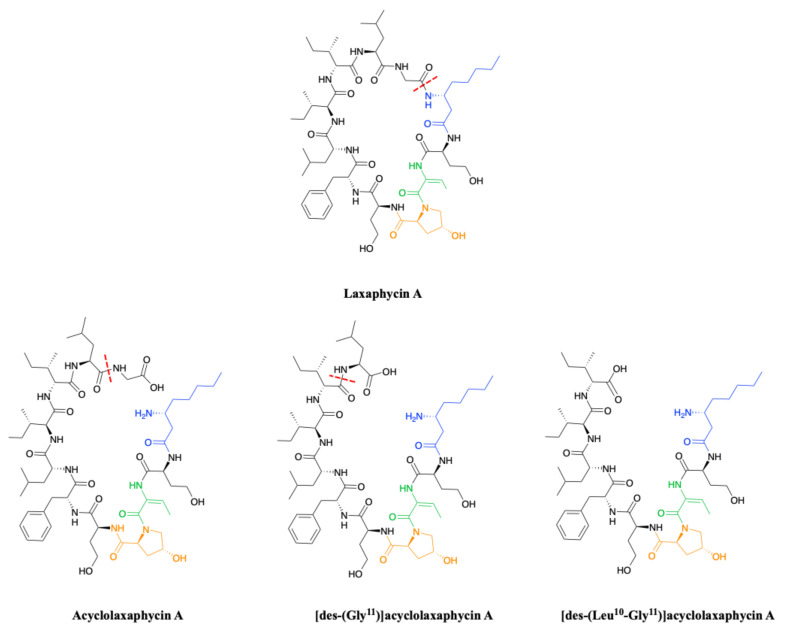
Structure of acyclic laxaphycins A compared to laxaphycin A. Amino acid deletion leading to the next acyclic laxaphycin A are indicated by red marks.

To complete this set of compounds, three acyclic laxaphycin A type peptides were discovered [27]. Acyclolaxaphycin A is equivalent to laxaphycin A opened between (3R)-Aoc in position 1 and glycine in position 11. NMR and MS spectra of [des-(Gly^11^)]acyclolaxaphycin A show a loss of glycine 11. The [des-(Leu^10^-Gly^11^)]acyclolaxaphycin A corresponds to acyclolaxaphycin A having lost glycine in position 11 and leucine in position 10 (Figure 2). Like their cyclic structural analogues, laxaphycin A2 and [d-Val^9^]laxaphycin A, these new acyclolaxaphycins A were not found in samples of *Anabaena* sp. collected by Bonnard et al. for their study on the toxicity of cyanobacterial blooms, thus highlighting that laxaphycin production might be seasonally affected or dependent on the environmental conditions [28].

**Figure 3:**
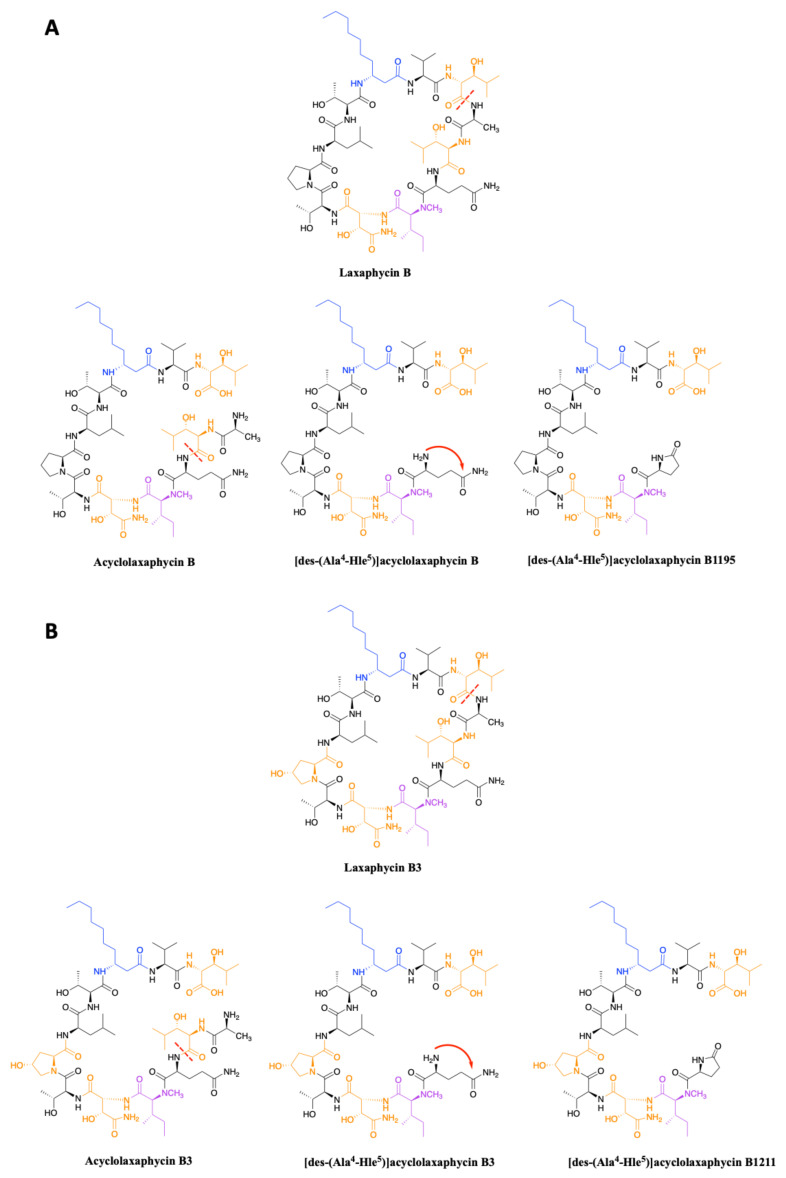
(**A**) Structure of acyclic laxaphycins B compared to cyclic laxaphycin B; (**B**) Structure of acyclic laxaphycins B3 compared to cyclic laxaphycin B3. Positions of ring opening or structural modifications leading to the next acyclic laxaphycins are designated by red marks.

In 2015, upon examination of an extract of *A. torulosa*, Bornancin et al. also found two acyclic laxaphycins, B and B3, that differ from their cyclic homologs by an *m/z* value 18 amu higher [32]. Furthermore, both peptides responded positively to the ninhydrin test, confirming an uncapped *N*-terminus. After fragmentation by MS-MS, the peptide is proposed to be open between 3-hydroxyleucine in position 3 and alanine in position 4. Stereochemistry is preserved for all amino acids between the cyclic and acyclic versions (Figure 3). These two new acyclolaxaphycins B were not found in other samples of Anabaena sp. collected at different locations in the lagoon of Moorea, as in the case of the new laxaphycins A mentioned above [28]. Recently, two acyclolaxaphycins were isolated from the sea hare *Stylocheilus striatus* that feeds on the cyanobacterium *A. torulosa*, producing laxaphycins [33]. High resolution mass spectrometry (HRMS) analysis allowed to propose [des-(Ala^4^-Hle^5^)]acyclolaxaphycins B and B3 that result from the loss of the two successive alanine and 3-hydroxyleucine in positions 4 and 5 (Figure 3). NMR analyses sustained this proposal due to the absence of correlations between alanine and a 3-hydroxyleucine. These two compounds undergo a spontaneous cyclization of the *N*-terminal glutamine to form a pyroglutamate, resulting in [des-(Ala^4^-Hle^5^)]acyclolaxaphycin B3 and B1211, respectively (Figure 3) [34].

### 2.2. Hormothamnin

**Figure 4:**
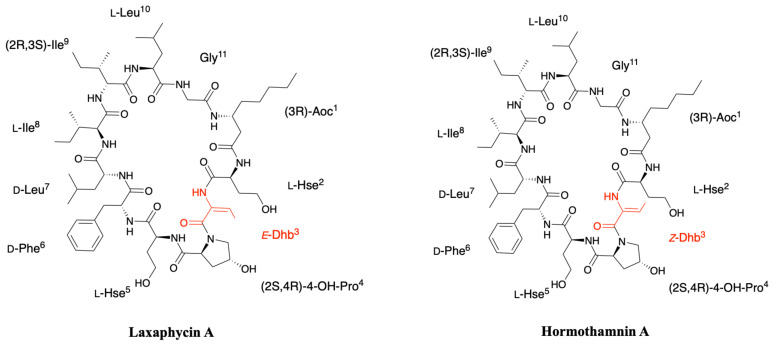
Structure of hormothamnin A compared to laxaphycin A. Changes between structures are marked in red.

The first known example of this peptide series was not termed laxaphycin but hormothamnin and was isolated by Gerwick’s group in 1989 from the cyanobacterium *Hormothamnion enteromorphoides*, collected in Playa de Luquillo in Puerto Rico [29]. About 15 hormothamnin peptides were detected in the crude extract but only one, hormothamnin A, the major compound and the most lipophilic one was fully characterized. Hormothamnin A contains the same 11 amino acids found in laxaphycin A sequence, albeit NMR analysis identified a *Z*-configuration for dehydrobutyrine (Figure 4) [35,36]. As it is the case for most of the compounds isolated from natural sources, this peptide was named accordingly to the organism from which it was extracted, however, as it was the only example among the 15 peptides detected in the producing organism to be fully characterized it failed to impose its name to the peptide family. 

### 2.3. Lobocyclamides

**Figure 5:**
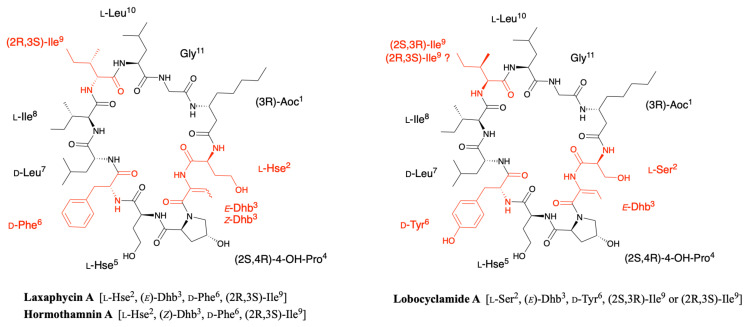
Structure of lobocyclamide **A** compared to laxaphycin **A** or hormothamnin **A**. Changes between structures are marked in red.

Lobocyclamides A, B, and C were isolated in 2002 from a sample of *Lyngbya confervoides* collected in Cay Lobos, Bahamas [30]. Lobocyclamide A differs from laxaphycin A by a serine in position 2 instead of an homoserine and a d-tyrosine that replaces the d-phenylalanine in position 6 (Figure 5). The article of MacMillan et al. suggests a l-*allo*-Ile in position 9 while indicating that it is certainly the same configuration as for laxaphycin A and hormothamnin A, i.e., a d-*allo*-Ile. It can be assumed that lobocyclamide A possesses a d-*allo*-Ile in position 9 and that an error was reported in the article. Differences are observed between the structure proposed in the figure and the text of the article. The figure suggests a Ser in position 2, a Hyp in position 4, a Hse in position 5, and a d-Tyr in position 6 while the text indicates a Hse, Pro, Ser, and d-Tyr in these respective positions. These slight differences in position and stereochemistry show the complexity that chemists face in confirming structures of natural products [9].

**Figure 6:**
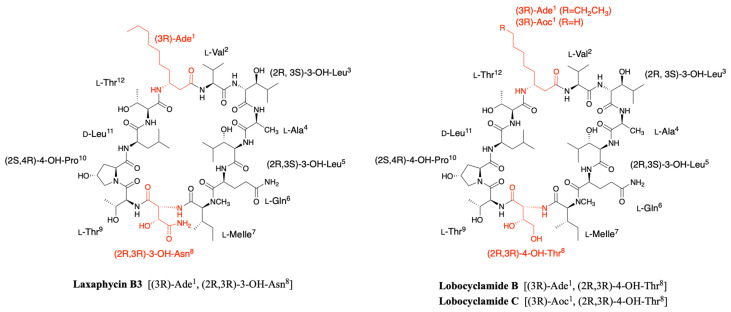
Structure of lobocyclamides B and C compared to laxaphycin B3. Changes between structures are marked in red.

Lobocyclamide B structure is very close to the previously reported laxaphycin B and B3 [30]. The main differences are constituted by a (2R,3R)-4-hydroxythreonine replacing the 3-hydroxyasparagine in position 8, which has never been reported in other peptides of the series, and a 4-hydroxyproline in position 10 that replaces the proline. The absolute configurations of the amino acids remain identical with those observed for laxaphycin B despite amino acid variations (Figure 6) [23]. 

The only difference observed between lobocyclamide B and C is the size of the long carbon chain present in position 1. Indeed, lobocyclamide C contains a β-aminooctanoic acid instead of a β-aminodecanoic acid, bringing it closer to the structure of laxaphycin D. 

### 2.4. Scytocyclamides

**Figure 7:**
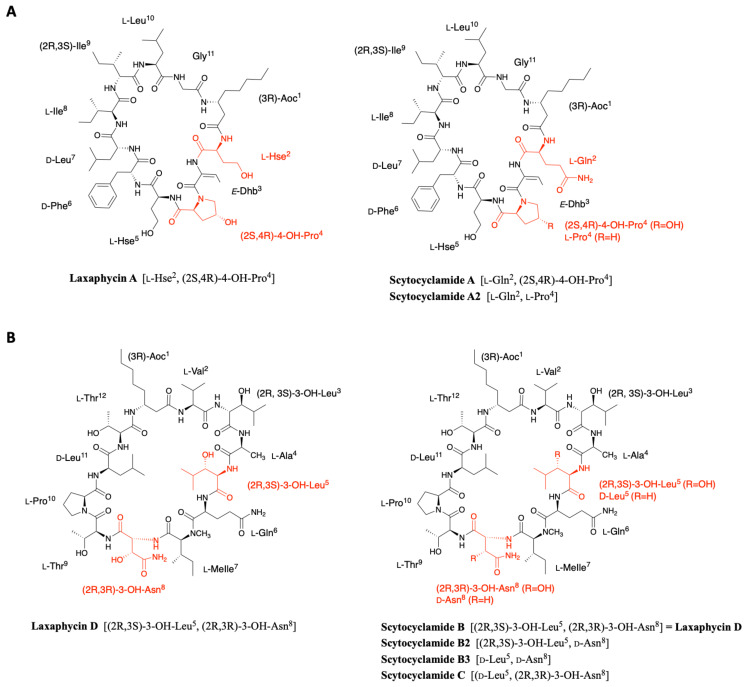
(**A**) Structure of scytocyclamides A and A2 compared to laxaphycin A; (**B**) Structure of scytocyclamides B, B2, B3, and C compared to laxaphycin D. Changes between structures are marked in red.

Scytocyclamides A, B, and C were first isolated from the cultivated fresh water *Scytonema hofmanni* PCC 7110 by Grewe in the early 2000s [37]. This initial study was recently completed by Sivonen’s team, who isolated three new scytocyclamides A2, B2, and B3 from the same cyanobacterium [38]. The analysis of the organic extract by ultra performance liquid chromatography allowed the isolation of six peaks, three corresponding to the scytocyclamides A-C previously described by Grewe and three less abundant peaks associated with masses suggesting less hydroxidic peptides. The combination of NMR, PSD spectra, MALDI-ToF mass spectrometry, and later MS-MS spectra of the scytocyclamide A-C showed that the amino acid sequence could be recovered from different generated ions in which the proline is always in the *N*-terminal position. The same fragmentation model was applied to the MS-MS spectra of the new scytocyclamides revealing their sequence and in particular the amino acids having lost a hydroxy group. Within this set of peptides, scytocyclamides A and A2 are related to laxaphycin A and scytocyclamides B, C, B2, and B3 to laxaphycin B. As the later article is focused on the biosynthesis of these peptides, amino acid stereochemistry was deduced from the epimerases found in the biosynthetic gene cluster and from analogy with peptides of the same family (Figure 7A).

Scytocyclamide A is a close analogue of laxaphycin A that differs only by the replacement of homoserine by a glutamine in position 2 [37]. Scytocyclamide A2 differs from scytocyclamide A by replacing 4-hydroxyproline with a proline in position 4 (Figure 7A) [38].

The scytocyclamide B sequence was determined by homology with analogues such as laxaphycin, hormothamnin, or lobocyclamide [37]. This comparison identified that scytocyclamide B has the laxaphycin D sequence (Figure 7B). In the case of scytocyclamide C, MALDI-ToF analysis shows a mass difference of 16 Da with scytocyclamide B, showing a loss of one hydroxy group in the sequence. The very strong similarities with the sequence of scytocyclamide B observed during the analyses accounted for the substitution of the 3-hydroxyleucine in position 5 by a leucine. Thus, the structure of scytocyclamide C is closely related to that of scytocyclamide B (laxaphycin D) (Figure 7B). Similarly, scytocyclamide B2 contains a d-asparagine while scytocyclamide B (laxaphycin D) contains a d-hydroxyasparagine in position 5 (Figure 7B) [38]. Finally, scytocyclamide B3 cumulates the two modifications present in scytocyclamides B2 and C with a d-asparagine in position 8 and a d-leucine in position 5 replacing the hydroxylated counterparts found in scytocyclamide B (Figure 7B). 

### 2.5. Lyngbyacyclamides

**Figure 8:**
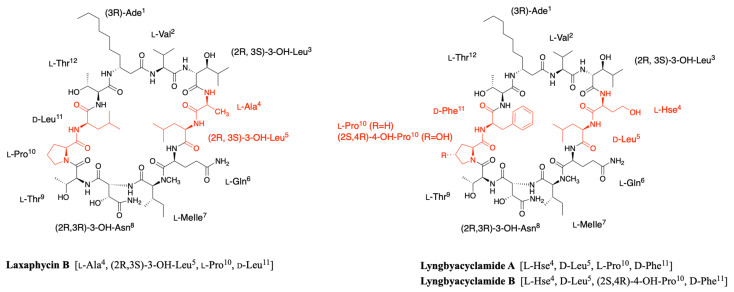
Structure of lyngbyacyclamides A and B compared to laxaphycin B. Changes between structures are marked in red.

In 2010, a marine cyanobacterium *Lyngbya* sp. collected at the Ishigaki Island in Japan allowed the isolation of two other members related to laxaphycin B, called lyngbyacyclamides A and B [39]. Thanks to nuclear Overhauser effect (NOE) and heteronuclear multiple bond correlations (HMBC) in NMR, the complete structure of both lyngbyacyclamides was established. Lyngbyacyclamide A differs from laxaphycin B by replacing l-alanine in position 4 by l-homoserine, (2R,3S)-3-hydroxyleucine in position 5 by d-leucine and d-leucine in position 11 by d-phenylalanine (Figure 8). Lyngbyacyclamide B has the same lyngbyacyclamide A sequence, except that it contains an 4-hydroxyproline instead of a proline in position 10 (Figure 8).

Stereochemistry of amino acids was partially deduced from Marfey’s analysis and comparison with analogous compounds within the laxaphycin B series. This was confirmed by the synthesis of this peptide a few years later, assuming that the non-proteinogenic amino acids were in the same absolute configuration as laxaphycin B [23]. Comparison of the NMR data of the synthetic peptide with the one reported for natural lyngbyacyclamide A confirmed that the stereochemistry observed within the laxaphycin family is generally conserved. 

### 2.6. Trichormamides

**Figure 9:**
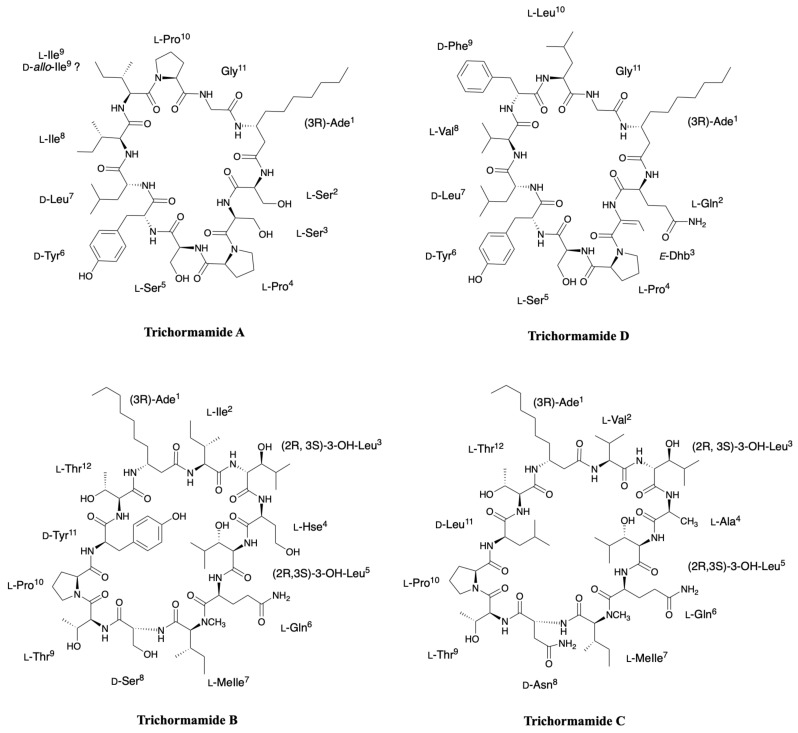
Structure of trichormamides A, B, C and D as described by Luo et al. [40,41].

In the context of research on natural antiproliferative products from freshwater cyanobacteria, Orjala’s group isolated four peptides, trichormamides A-D, from two different cyanobacteria, *Trichormus* sp. UIC 10339 collected in Raven Lake (WI, USA) and *Oscillatoria* sp. UIC 10045 collected in Downers Grove (IL, USA) [40,41]. Trichormamide A complete structure was determined thanks to NMR spectra and was defined as an undecapeptide related to laxaphycin A. Mass fragmentation analysis allowed to fragment the cyclic peptide, with a first cleavage at the *N*-terminal side of proline, and to confirm a structure with substantial differences with laxaphycin A as almost 60% of the amino acid differs between the two peptides, making it the most distant analogue in terms of sequence identity from laxaphycin A. It is worth noting that trichormamide A is the only example in the laxaphycin A-type peptides lacking the dehydrobutyrine residue. Marfey’s analysis enables to assign the absolute configuration of amino acids in the sequence and prove once again the consistency of the series (Figure 9). Trichormamide D is a laxaphycin A analogue isolated from *Oscillatoria* sp. UIC 10045 strain [41]. Rotating frame Overhauser effect spectroscopy (ROESY) correlations enable to confirm an *E*-configuration for the dehydrobutyrine residue. Glutamine, proline, valine, and serine were determined as l-configured while tyrosine, phenylalanine, and 3-aminodecanoic acid are d-configured. The stereochemistry of the two leucines were more difficult to attribute but were finally assigned as L for leucine in position 10 and D for leucine in position 7, which is consistent with stereochemistry found in laxaphycin A analogues (Figure 9).

Trichormamides B and C are 12-residue laxaphycin analogues isolated from Trichormus sp. UIC 10339 and from *Oscillatoria* sp. UIC 10045, respectively [40,41]. While the only difference between trichormamide C and laxaphycin B is the replacement of the (2R,3R)-3-hydroxyasparagine in position 8 by a simple d-asparagine, the trichormamide B contains three modifications with respect to laxaphycin B (Figure 9). For trichormamide B, the valine in position 2 is replaced by an isosteric isoleucine, the alanine in position 4 by a homoserine and the d-leucine in position 9 by a d-tyrosine. The most striking modification concerns the substitution of the 3-hydroxyasparagine in position 8 by a d-serine that made it a unique representant in this laxaphycin B series.

Some of the modifications contained in the trichormamides made them an attractive group for total synthesis in order to establish further structure activity relationships or simple structure confirmation. For this purpose, trichormamide A was synthesized in 2018 as described by Luo et al. [40], along with a diastereoisomer containing a d-*allo*-Ile in position 9 instead of the described l-Ile [42]. Indeed, laxaphycin A and its analogues contain a d-*allo*-Ile in position 9, suggesting an error in the structure of trichormamide A described with an l-Ile. A difference was observed in the resolution and width of the peaks obtained in NMR for the synthetic trichormamide A and the described natural peptide. However, this difference is maintained between the natural peptide and the diastereoisomer containing a d-*allo*-Ile, making it impossible to conclude on the stereochemistry of this amino acid. Furthermore, due to the lack of supply of the natural peptide, comparison between natural and synthetic products was impossible. Trichormamide C was synthesized in 2020 and its structure was confirmed by comparison of NMR spectra of synthetic and natural peptides [43].

### 2.7. Heinamides

**Figure 10:**
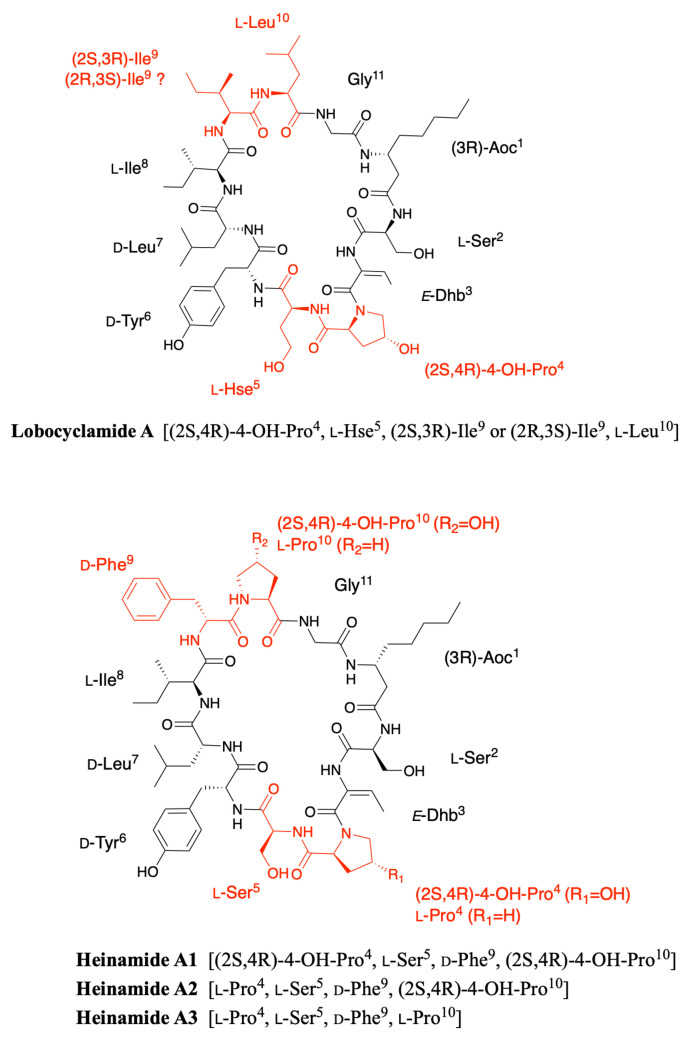
Structure of heinamides A1-A3 compared to lobocyclamide A. Changes between structures are marked in red.

**Figure 11:**
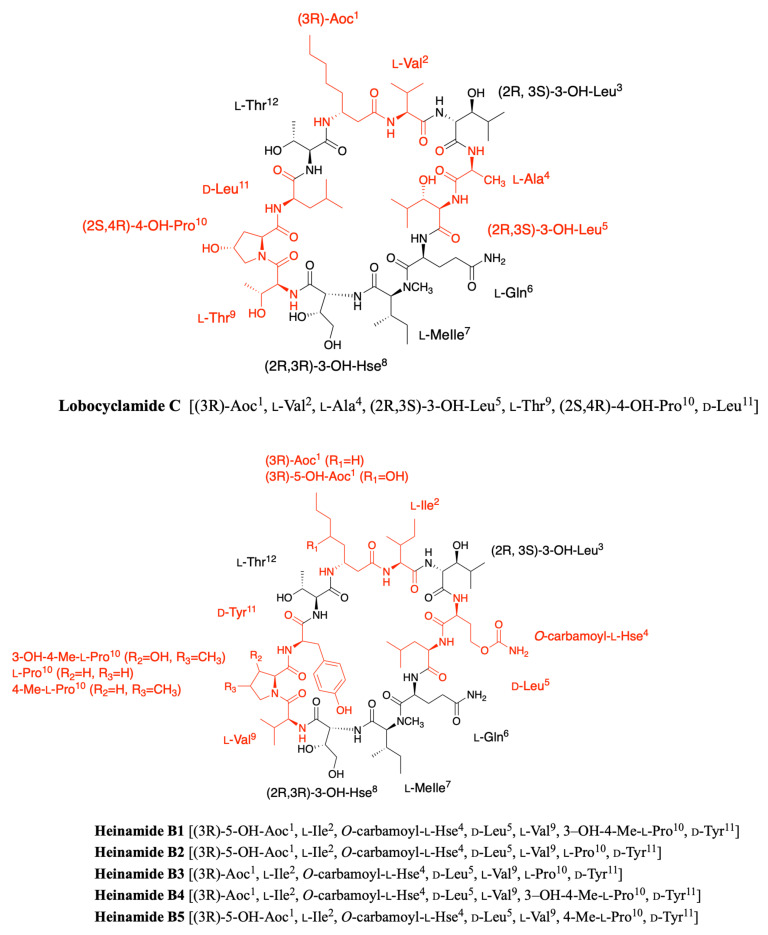
Structure of heinamides B1-B5 compared to lobocyclamide C. Changes between structures are marked in red.

Very recently, Sivonen’s group identified eight new laxaphycins from *Nostoc* sp. UHCC 0702 (collected from lake Villähteen Kukkanen) and named heinamides, three of A-type and five of B-type laxaphycins [44]. By cultivating on ^15^N-containing medium and comparing the masses of the labeled and unlabeled compounds, they showed that heinamides A1, A2, and A3 contain 11 nitrogen atoms, as do the A-type laxaphycins. In contrast, heinamides B1, B2, B3, B4, and B5 contain 14 nitrogen atoms, one more than B-type laxaphycins. By recombination of pure and mixed heinamides LC-MS/MS and NMR data, structures for heinamides type A and B could be proposed (Figure 10 and Figure 11).

In the case of heinamides A, no major changes were observed compared to the different laxaphycins A (Figure 10). On the other hand, heinamides B have particular features that make them quite unique (Figure 11). At position 1, 3-aminooctanoic acid is hydroxylated on carbon 5 with undescribed stereochemistry, whereas this modification has never been reported before. Position 4 is a homoserine, already observed in several laxaphycins B, but this time containing a carbamoyl group on the alcohol of the side chain, explaining the additional nitrogen atom. This type of amino acid has never been found in other natural peptides. The proline at position 10 can be hydroxylated on carbon 3 and methylated on carbon 4, depending on the different heinamides. The stereochemistry of these two carbons has not been determined. The 3-OH-4-Me-Pro is not much described in the case of natural products from cyanobacteria, but is found in fungi as in the case of echinocandins [45]. Finally, a (2R,3R)-4-OH-Thr occupies position 8 as previously described in lobocyclamide B and C [30]. However, in view of the data obtained in the study of heinamide biosynthesis, it would seem that it is in fact a homoserine hydroxylated on carbon 3 rather than a threonine hydroxylated on carbon 4 [44]. Therefore, this amino acid will be named (2R,3R)-3-OH-Hse in the rest of this review, although this does not change the final structure.

To date, 13 different undecacyclic peptides belonging to the laxaphycin A family (Table 1, Figure S1), and 21 dodecacyclic peptides in the laxaphycin B family are known (Table 2, Figure S2). Three acyclic laxaphycin A and six acyclic laxaphycin B completed these two groups. Cyclic and acyclic peptides were isolated from marine, freshwater, or terrestrial cyanobacteria and even from a marine mollusk found at different locations all around the world.

Laxaphycin A analogues are relatively homogeneous in terms of sequence, with the notable exception of the trichormamide and heinamide analogues that share less than 50% of their amino acids in common (Table 1).

One of the most frequent modifications observed in laxaphycin B relies on the hydroxylation state. A total 70% of the analogues are concerned by this modification type, affecting the (2R,3S)-3-hydroxyleucine in position 5, the (2R,3R)-3-hydroxyasparagine in position 8, and the proline in position 10 that are often replaced by d-leucine, the d-asparagine or (2S,4R)-4-hydroxyproline, respectively. Alanine 4 is also concerned and mostly replaced by a homoserine. Among the other observed substitution, valine in position 2 is replaced by its β-branched equivalent isoleucine, whereas d-leucine in position 11 is changed by isosteric amino acids such as d-phenylalanine or d-tyrosine. Thus, most of the time, amino acid variations are made with isosteres, but stereochemistry remains preserved (Table 2). However, the determination of structures and stereochemistry is often complex in the case of natural products, with overlapping or similar signals. Thus, wrong attributions can be made, so that one cannot be sure of the proposed structures until the total synthesis is performed. About 20 of these misassignments have been reported in 2015 on various natural products and laxaphycin does not constitute an exception [24]. In the next part of this review, we describe and summarize the efforts that have been pursued to synthesize the laxaphycins.

## 3. Chemical Synthesis

**Figure 12:**
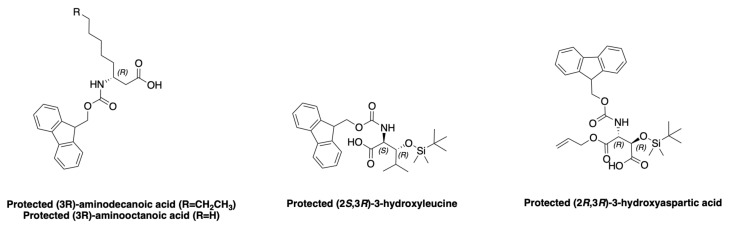
Structure of protected non-proteinogenic amino acids needed for the laxaphycin B synthesis.

Cyclic peptides have gained interest in recent years, particularly because of their resistance to degradation by proteases [46]. Most cyclic peptides have low membrane permeability, but some are able to penetrate the cell membrane, such as cyclosporine [47]. Although many marine peptides are isolated from various organisms, few are studied in clinical research. In the case of cyclic peptides isolated from cyanobacteria, none have been approved as drugs [48,49]. Various reasons can be given for this lack of success, such as the number of steps to be completed in the laboratory beforehand. Indeed, the minute quantities of peptides isolated directly from various organisms preclude their study. It is therefore up to the chemists to produce them on a larger scale. However, it is necessary to develop efficient synthesis methods to obtain these complex molecules. Among the peptides cited in the first part of this review, laxaphycin B was the first to be fully synthesized [23] along with a very few analogues. In all the cases, the syntheses of the non-classical amino acids included in their sequences are required. This prerequisite concerns the (2S,3R)-3-hydroxyleucine stereoisomers, the 3-aminodecanoic acid, the 3 amino-octanoic acid, and the (2R,3R)-3-hydroxyaspartic acid (Figure 12). Furthermore, for each amino acid, relevant protections of the functionality are needed to develop the solid phase peptide synthesis scheme. In this section, we describe the synthetic methods used to obtain these different amino acids, as well as the complete syntheses of some laxaphycins and their analogues. The possible synthesis techniques for obtaining dehydrobutyrine are also described, although it is possible to obtain it directly through dehydration of threonine.

### 3.1. Synthesis of Characteristic Amino Acids in Laxaphycins

#### 3.1.1. Synthesis of 3-Aminodecanoic acid and 3-Aminooctanoic acid

**Figure 13:**
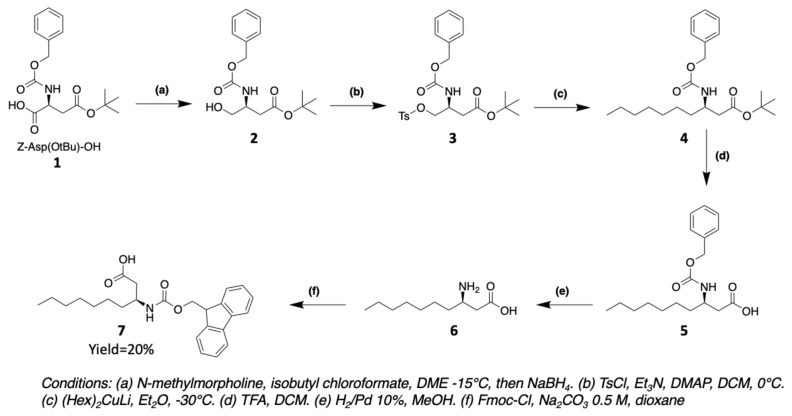
Synthesis of protected (3R)-aminodecanoic acid.

(3R)-aminodecanoic acid (Ade) **7** is obtained from commercially protected aspartic acid **1** following the synthesis method of Buron et al. [50]. The carboxylic acid function of *Z*-Asp(OtBu)-OH **1** is converted to anhydride with isobutyl chloroformate. The unsymmetrical anhydride is then reduced to alcohol **2** using NaBH_4_. The alcohol is transformed to a tosyl group that allows a nucleophilic substitution with an organocuprate (Hex)_2_CuLi to introduce the fatty chain. After successive deprotection of the acid and amine groups, the latter is protected by a Fmoc group in order to use it for solid phase during peptide synthesis (Figure 13). (3R)-aminooctanoic acid (Aoc) is equivalently synthesized using the organocuprate corresponding to the desired chain.

#### 3.1.2. Synthesis of Hydroxylated Amino Acids

**Figure 14:**
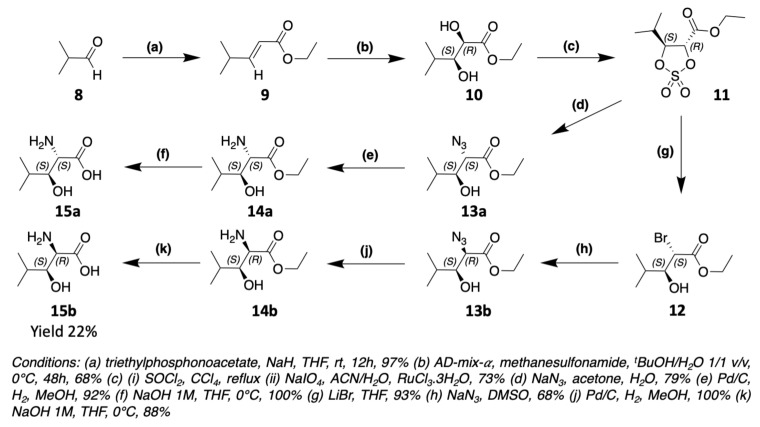
Synthesis of (2R,3S)-3-OH-Leu by Sharpless dihydroxylation.

The biggest challenge encountered by chemists to obtain β-hydroxylated amino acids is the control of the stereochemistry of the asymmetric carbons. It is therefore necessary to go through very stereoselective reactions. The synthesis of (2R,3S)-3-OH-Leu, found notably in laxaphycin B and lobocyclamide B, has been reported in the literature. For the total synthesis of laxaphycin B, Boyaud et al. used Sharpless dihydroxylation to obtain (2R,3S)-3-OH-Leu (Figure 14) [23,51]. This method was notably used by Hale et al. for the synthesis of the antibiotic depsipeptide A83586C [52]. The first step is a Wittig reaction between isobutyraldehyde **8** and triethylphosphonoacetate to form the corresponding alkene **9**. Next, **9** is dihydroxylated into **10** by the action of AD-mix-α and methane sulfonamide. The alcohols groups are protected as a cyclic sulfite, later oxidized to cyclic sulfate **11**. Sodium azide reacts according to a nucleophilic substitution on the alpha carbon of **11** and the alcohol in the beta position is recovered by hydrolysis. The N_3_ group of **13a** is then reduced in NH_2_ function to obtain (2S,3S)-3-OH-Leu **15a**. To produce (2R,3S)-3-OH-Leu **15b**, an additional step is necessary to modify the stereochemistry. LiBr reacts on the cyclic sulfate **11** in alpha to reverse the stereochemistry of carbon 2. The addition of NaN_3_ on **12** will then recover the desired stereochemistry to obtain the (2R,3S)-3-OH-Leu **15b** after reduction of N_3_ to NH_2_ function. 

**Figure 15:**
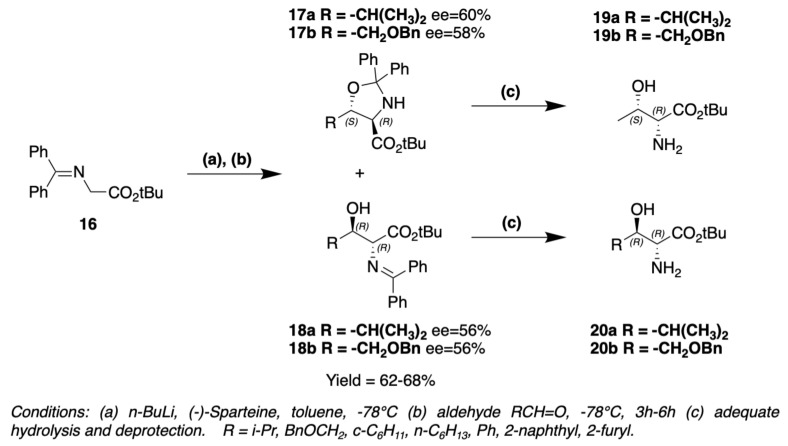
Synthesis of 3-hydroxyleucine and 3-hydroxyhomoserine by an aldolization reaction.

Absolute configuration of 3-hydroxyleucine present in lobocyclamide B was determined through a synthesis involving an aldolization reaction (Figure 15) [53]. *N*-(diphenylmethylene)glycine *tert*-butyl ester **16** is transformed into its enolate by the action of *n*-BuLi. The addition of enolate to isobutyraldehyde is assisted by sparteine. Two products are obtained, *threo*-oxazolidine **17a** and *erythro*-imine **18a**, leading to (2R,3S)-3-OH-Leu **19a** and (2R,3R)-3-OH-Leu **20a** after hydrolysis, respectively. *threo*-oxazolidine and *erythro*-imine could be obtained in different proportions depending on the base used to form the enolate, *n*-BuLi/sparteine or LDA [53]. MacMillan et al. used the same method to obtain 4-hydroxythreonine **19b**–**20b** (or 3-hydroxyhomoserine) with the addition of enolate to *O*-benzylglyoxal (Figure 15) [53]. This reaction could be generalized for different β-hydroxylated amino acids, if the right aldehyde is used to create the side chain of the amino acid (Figure 15).

**Figure 16:**
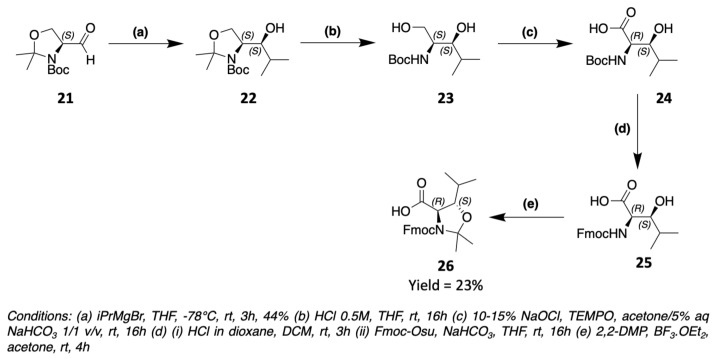
Synthesis of (2R,3S)-3-OH-Leu using Garner’s aldehyde.

These two examples are the only ones reported to date in the context of the synthesis of laxaphycins and their derivatives. However, 3-hydroxyleucines are found in other peptides, and it is possible to obtain them by different synthetic routes. The (2R,3S)-3-OH-Leu present in skyllamycin is obtained via the Garner aldehyde of S configuration **21** (Figure 16) [54]. A Grignard reaction takes place between **21** and isopropylmagnesium bromide to form the corresponding secondary alcohol **22**. The oxazolidine is then hydrolyzed to obtain a primary alcohol **23** which is oxidized to carboxylic acid **24**. The amine can then be deprotected from its Boc to be reprotected by a Fmoc group, more suitable for peptide synthesis. The oxazolidine **26** is then reformed between the amine and the alcohol function in the beta position.

**Figure 17:**
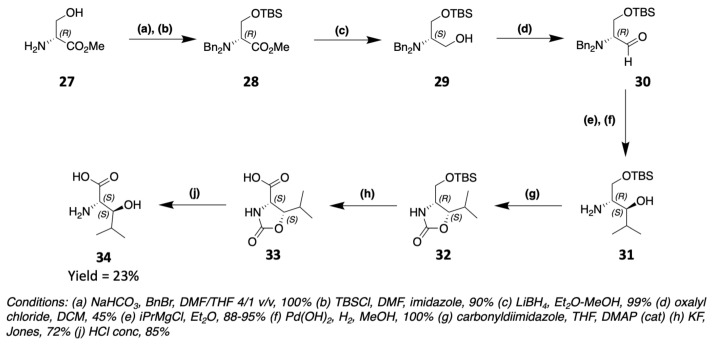
Synthesis of (2S,3S)-3-OH-Leu using chiral derivative of serine.

Muraymycin C1 [55] as well as alkaloid cyclopeptides [56] contain (2S,3S)-3-OH-Leu obtainable from a protected serine (Figure 17). The ester of the protected serine **28** is reduced to primary alcohol **29**, which is subsequently oxidized to aldehyde **30**. By a Grignard reaction of isopropylmagnesium bromide on **30**, the corresponding secondary alcohol **31** is obtained. After deprotection of the amine, an oxazolidinone **32** is formed between the amine and the secondary alcohol. The primary alcohol of the serine side chain can then be oxidized to carboxylic acid and **33** is hydrolyzed to give (2S,3S)-3-OH-Leu **34**. Several other synthesis routes can be used to obtain these compounds and are referenced in the literature [57,58,59,60].

**Figure 18:**
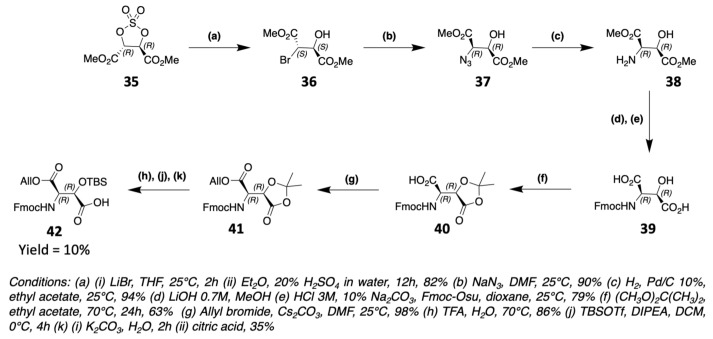
Synthesis of (2R,3R)-3-OH-Asp from dimethylester tartrate.

As part of the synthesis of laxaphycin B, Boyaud et al. also had to synthesize 3-hydroxyaspartic acid to graft its side chain on Rink Amide resin [61]. The synthesis starts with a cyclic sulfate **35** derived from dimethylester tartrate (Figure 18). The opening of **35** with LiBr gives access to a secondary alcohol and a bromine group. The bromine is then replaced by an azide to give **37**. The azide is reduced to an amine, which is then protected by an Fmoc group. The two ester functions of **38** are hydrolyzed to carboxylic acid. An acetonide **40** is formed between the carboxylic acid and the alcohol of the same carbon in order to be able to differentiate the two carboxylic acid groups. The remaining acid can thus be protected by an allyl group, allowing later the hydrolysis of the acetonide and the protection of the alcohol in beta position providing **42**. This type of synthesis has been used by other groups to obtain 3-hydroxyaspartic acid [62] or 3-hydroxyasparagine [63]. 

The aminohydroxylation of Sharpless is also used to obtain 3-hydroxyasparagine or 3-hydroxyaspartic acid in the synthesis of other peptides such as polytheonamide B [64] or ramoplanin A2 [65]. It is also possible to obtain these compounds via the opening of an oxazoline [66].

**Figure 19:**
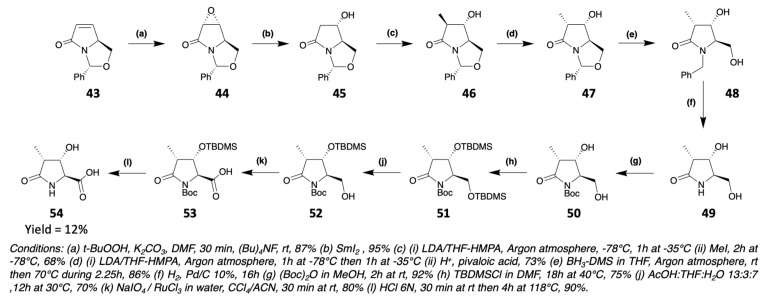
Synthesis of (2S,3S,4S)-3-hydroxy-3-methylproline from γ-lactam.

Echinocandins are natural peptides found in different ascomycota fungi. They notably contain a 4-OH-Pro and a 3-OH-4-Me-Pro. The 3-OH-4-Me-Pro, which is also found in heinamides, can be synthesized in several ways [67], in particular by starting from a γ-lactam **43** prepared from pyroglutaminol (Figure 19) [68]. First, **43** is diastereospecifically transformed into epoxide **44**. The oxirane is then cleaved to release a secondary alcohol. After a deprotonation/methylation/reprotonation sequence, the cis-disubstituted lactam **47** is obtained. Then, **47** is reduced to trisubstituted *N*-benzyl pyrrolidine **48**. The benzyl group is removed by hydrogenolysis and replaced by a Boc protection giving **50**. The two alcohol functions can thus be protected by a TBDMS group, which will be selectively removed from the primary alcohol only providing **52**. The latter can then be oxidized by the Sharpless method [69] and the (2S,3S,4S)-3-hydroxy-4-methylproline **54** is recovered by acid hydrolysis (Figure 19).

#### 3.1.3. Synthesis of Dehydro Amino Acids

Dehydroamino acids are commonly found in cyanobacterial peptides [9] and more generally in peptides of microbial origin [70]. Several studies have shown that these amino acids can structure peptides, similarly to 2-aminoisobutyric acid (Aib) [71,72]. In view of their role in the structure of peptides, it is important to understand how they are biosynthesized and also to develop their syntheses for an efficient peptides’ production [73]. In this sub-section, we will develop the synthesis pathways used to obtain dehydrobutyrine, present in type A laxaphycins. 

Dehydroamino acids are mainly obtained through the dehydration of their β-hydroxylated equivalent amino acid, i.e., threonine for dehydrobutyrine, by an elimination mechanism. Indeed, attempting to insert dehydrobutyrine directly into a peptide sequence brings very low yield because of the low nucleophilicity of the enamine that impairs its coupling with other amino acids. It is therefore more interesting to insert a threonine into the peptide sequence and then dehydrate it, either once the peptide elongation is completed, or on a smaller sequence of the peptide which will then be inserted into the rest of the sequence. This dehydration reaction has been widely reported in the literature [74,75]. We report here some cases of dehydration to obtain dehydrobutyrine.

**Figure 20:**
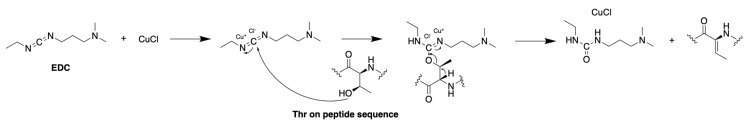
Proposed mechanism of action of EDC on threonine to obtain dehydrobutyrine [76].

Albericio’s group has developed a synthesis method, using EDC/CuCl for the dehydration of threonine, which can be applied both in solution and in solid phase synthesis (Figure 20) [76,77]. The elimination mechanism would be by an E1 or E1cb, with EDC activating the alcohol of the threonine in the presence of CuCl in a DMF/DCM mixture followed by β-elimination of the activated group. 

The advantage of this technique is that these reagents are inexpensive and simple to use, so the reaction is performed under mild conditions at room temperature. In addition, only the most thermodynamically stable *Z-*isomer of Dhb is produced. In the case of dehydration of a fragment of the peptide, a solution reaction with EDC/CuCl (2:1.2) in solution in DCM/DMF is preferred for a relatively short time (about 20h). For dehydration of the whole peptide, a solid support reaction with EDC/CuCl (2:1.2) in solution in DCM/DMF for a much longer time (several days) is used.

In the case of the synthesis of hormothamnin A, the dehydration reaction was done directly on solid support, but with DIC/CuCl reagents [78]. Indeed, EDC can cause secondary reactions, such as the formation of a cyclic by-product (*O*-alkyl *N*-acylisourea) [76] or guanidylation of the *N*-terminal amino acid, the Fmoc group being sensitive to EDC. Dehydration was therefore performed once the complete linear peptide was obtained, with DIC/CuCl (100:3 equiv) in a DCM/DMF mixture (1:1) for 7 days.

**Figure 21:**
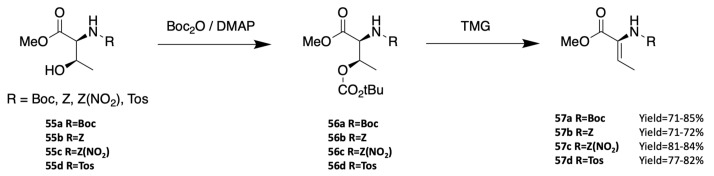
Dehydration of bi-protected threonine by the action of Boc_2_O/DMAP and then TMG base [80].

Ferreira et al. developed a dehydration method using (Boc)_2_O/DMAP to protect the side chain alcohol of threonine **55** and the *N,N,N*-tetramethylguanidine base (TMG) to generate removal of the protected alcohol of **56** (Figure 21) [79,80]. This method is stereoselective and yields the *Z*-isomer of the dehydro amino acid **57**. Tian’s team used the same method to generate dehydrobutyrine one-pot from the threonine already included in the peptide sequence [81].

**Figure 22:**
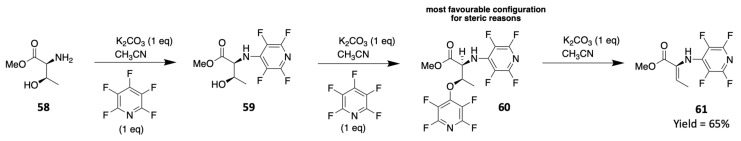
Dehydration of mono-protected threonine by the action of K_2_CO_3_ base and pentafluoropyridine.

Still with an E1cb elimination reaction, Webster et al. showed that it was possible to dehydrate threonine **58** in the presence of pentafluoropyridine and K_2_CO_3_ base (Figure 22) providing protected dehydrobutyrine **61** [82]. 

**Figure 23:**
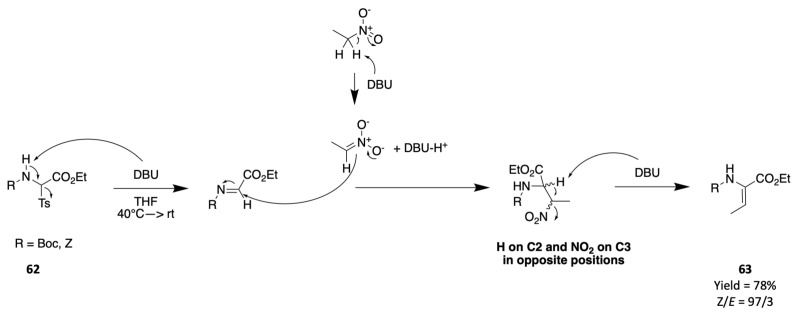
Obtention of dehydrobutyrine from alpha-protected glycine in the presence of DBU base.

Nagano et al. reported an approach for obtaining dehydroamino acids **63** starting from alpha-tosyl glycine **62** and various nitroalkene in the presence of DBU as a base (Figure 23) [83]. 

To obtain dehydroamino acids other than dehydrobutyrine, other techniques than dehydration are possible and have been reported, notably in a review by Bonauer et al. [84]. 

### 3.2. Synthesis of Laxaphycin B-Type Peptides

**Figure 24:**
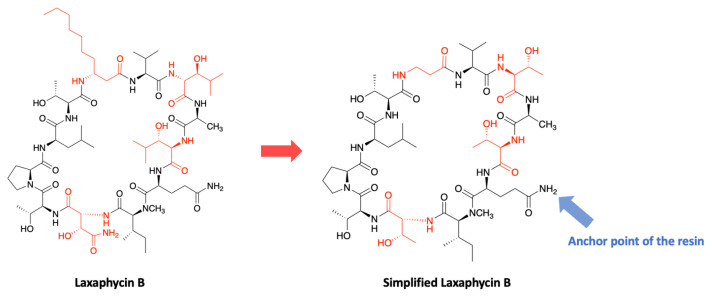
Structure of a simplified laxaphycin B used for the development of laxaphycin B synthesis.

In order to develop a strategy for the laxaphycin B synthesis, the structure has been simplified to contain only natural amino acids. The 3-hydroxyasparagine and the two 3-hydroxyleucines have been replaced by threonines with the same absolute configuration, and the β-aminodecanoic acid has been replaced by a β-alanine (Figure 24). It is important to note that at the time of the development of this synthesis, the two 3-hydroxyleucines were considered as diastereoisomers and were therefore replaced accordingly by a d-threonine and an l-threonine.

The first trials for the gain of laxaphycin B were based on a head-to-tail cyclization strategy on the solid support to avoid the isolation and purification of an intermediate linear peptide and the risk of dimerization. This cyclization method could be used because the peptide sequence contains an aspartic and a glutamic acid available to be anchored on the resin with their side chain, while the acid group is protected with an allyl. Depending on the resin choice, the aspartic and glutamic acid are recovered in their native form in the case of a Wang resin or are transformed into asparagine or glutamine if the synthesis is performed on a Rink amide MBHA resin.

**Figure 25:**
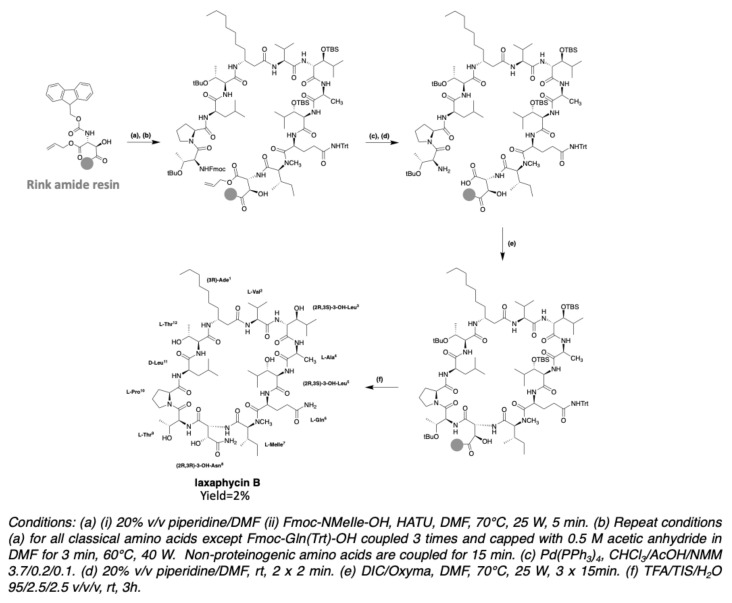
Optimized synthesis of laxaphycin B.

After preliminary assays, the strategy based on a cyclization between the glutamine and the (*N*)-methylisoleucine was discarded due to the lack of efficiency of the coupling reaction between these two amino acids. The anchoring point of the resin therefore had to be modified, by attaching the side chain of aspartic acid rather than glutamic acid. The elongation of the linear peptide was carried out with HATU/DIEA as coupling reagents to facilitate couplings, especially between the glutamine and the (*N*)-methylisoleucine. After the elongation of the peptide, the carboxylic acid of glutamine and the amine of threonine in position 9 were deprotected and subsequently engaged in the cyclization step with DIC/Oxyma. After side chain protections removal under acidic conditions, the simplified laxaphycin B analogue was obtained. Next, this methodology was applied to laxaphycin B. The replacement of β-alanine by β-aminodecanoic acid resulted in a slight decrease in coupling without making synthesis more problematic. Similarly, the addition of 3-hydroxyasparagine and 3-hydroxyleucine instead of simple asparagine and threonine was not a problem. Laxaphycin B could therefore be obtained by this optimized synthesis method and the LC-MS analysis gave an *m/z* of 1395.8 for [M + H]^+^ ion. In order to confirm the structure of the synthetic laxaphycin B, HPLC and NMR spectra were compared with the one of the natural laxaphycin B. Significant differences were observed for the proton’s chemical shifts, but also the retention time of the two molecules were different. Comparison of the amino acid stereochemistry found among the laxaphycin analogues allows to rapidly identify that the 3-hydroxyleucine in position 3 has a stereochemistry (2R,3S) in lobocyclamide B, lyngbyacyclamide B, as well as in laxaphycin B2, whereas it had been determined to be (2S,3S) in laxaphycin B. Re-evaluation of Marfey’s analyses showed that laxaphycin B contains two 3-hydroxyleucines in (2R,3S) configuration. Laxaphycin B was therefore re-synthesized considering these new stereochemistries and then compared to the natural molecule (Figure 25). Identical signals in HPLC and NMR were observed for both natural and synthetic laxaphycin B, confirming this new structure. The synthesis was then applied to lyngbyacyclamide A, allowing to confirm the stereochemistry of non-proteinogenic amino acids which were postulated by the group of Daisuke Uemura [39].

**Figure 26:**
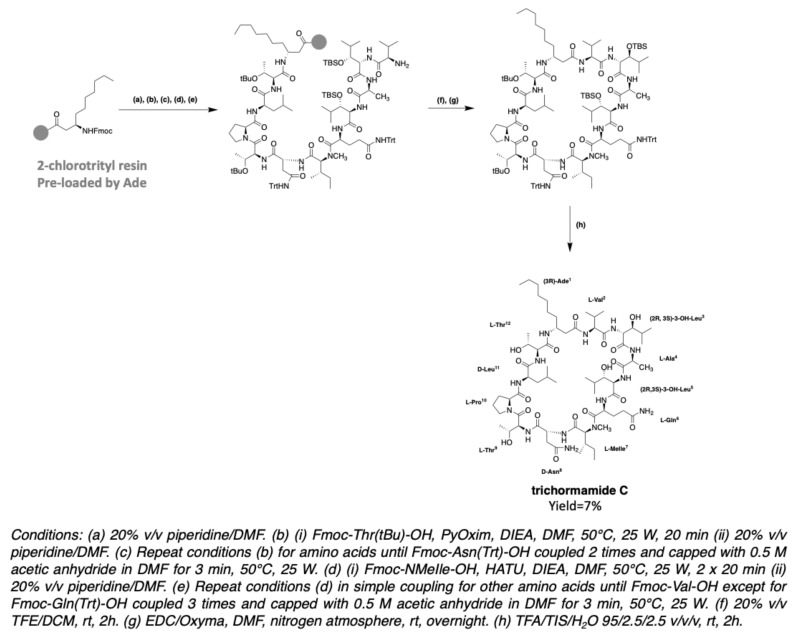
Optimized synthesis of trichormamide C.

In 2020, the synthesis of trichormamide C was undertaken by the same group [43]. Trichormamide C contains a d-asparagine instead of a (2R,3R)-3-hydroxyasparagine in laxaphycin B. Since this peptide contains 3-aminodecanoic acid (Ade), just like laxaphycin B, it was interesting to develop a method of synthesis allowing to use a minimal amount of Ade as its synthesis needs six steps for an overall yield of 20% (Figure 13). An attractive strategy was to graft it first onto a 2-chlorotrityl resin, allowing to recover the uncoupled part [85]. Unlike laxaphycin B, the synthesis temperature is lowered from 50 °C to 70 °C to avoid cleavage of the peptide from the chlorotrityl resin [86] and HATU was conserved as coupling reagent. Surprisingly, after soft peptide cleavage (20% TFE/DCM solution) to keep side chain protections, LC-MS analysis of the reaction crude revealed the guanidylation of threonine at position 9. DIC/Oxyma being less prone to this type of secondary reaction, were used for the whole synthesis instead of HATU/DIEA. However, the phenomenon worsened, showing similar reaction with DIC on every amino acid from Ade in position 1 to (*N*)-methylisoleucine in position 7. This observation suggests a particular structure for the beginning of the sequence, preventing the coupling from taking place correctly. To overcome this problem, PyOxim/DIEA was chosen, as these coupling reagents cannot produce guanidinium ions. HATU was kept for the coupling of the remaining amino acids from glutamine, being more suitable for this type of coupling. The use of PyOxim increases the yield and avoids the guanidylation side reaction. The use of a chlorotrityl resin grafted by Ade does not allow a cyclization directly on the resin, as in the case of the synthesis of laxaphycin B. This is why the peptide is cleaved from the resin with maintenance of the side chain protections. Cyclization is performed under pseudo-high dilution conditions with EDC/Oxyma as coupling reagents [87]. After final deprotection of the side chains, trichormamide C was obtained (Figure 26). HRMS analysis gave a *m/z* of 1401.8316 for [M+Na]^+^ ion while the calculated *m/z* is 1401.8333. This method was successfully applied to two other synthetic analogues containing d-leucines or d-threonines instead of the two 3-hydroxyleucines, showing the flexibility of this new synthesis. Obtaining trichormamide C by chemical means allowed comparison with the natural molecule previously reported by Luo et al. [41]. Although the natural compound was not available, the NMR data comparison showed similar values, suggesting a correct attribution of the structure of the natural peptide.

It should be noted that synthetic analogues of trichormamide C were obtained using only PyOxim/DIEA as coupling agents and not a mixture of PyOxim/DIEA and then HATU/DIEA, making the synthesis simpler despite a very slight decrease in the coupling of glutamine to *N*-methylisoleucine [88].

### 3.3. Synthesis of Laxaphycin A-Type Peptides

**Figure 27:**
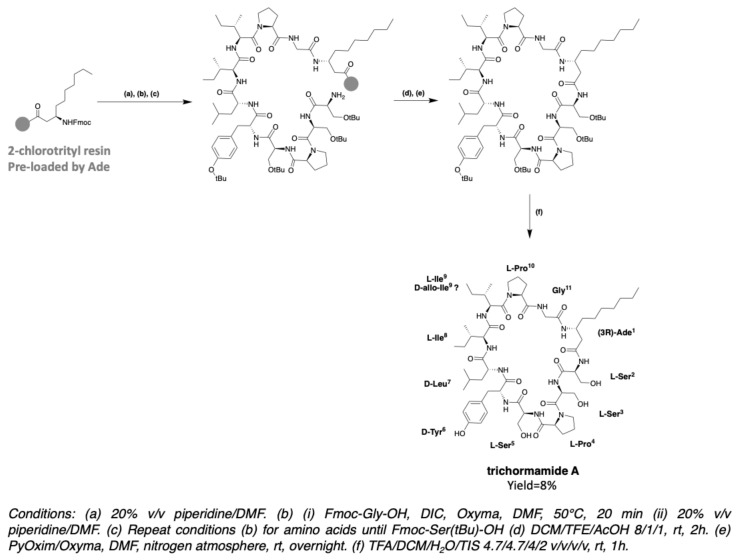
Optimized synthesis of trichormamide A.

The synthesis of trichormamide A was investigated in 2018, in order to develop a synthesis method that can be applied to the peptide of the laxaphycin A family [42]. In fact, trichormamide A is a model of choice for the development of this synthesis because it does not contain complex amino acids such as dehydrobutyrine, as other peptides have. Trichormamide A contains the β-aminodecanoic acid (Ade), whose chemical synthesis was already described above. All other amino acids are commercially available. As in the case of trichormamide C, Ade has been preloaded on a 2-chlorotrityl chloride resin. Synthesis was performed at 50 °C under microwave irradiations, using DIC/Oxyma coupling agents. The linear peptide was cleaved from the resin by the action of a DCM/TFE/AcOH mixture, while keeping the side chain protections. Cyclization was initiated in solution, under high dilution conditions, in the presence of PyOxim/Oxyma coupling agents, giving good results on this peptide sequence. The cyclic peptide was purified before deprotection of the side chains under strong acidic conditions (Figure 27). HRMS analysis gave an *m/z* of 1184.6919 for [M + H]^+^ ion while the calculated *m/z* is 1184.6931. The development of the synthesis of this peptide paves the way to obtain other analogous peptides, which would allow the structures to be verified and used for SAR studies. In this article, Gaillard et al. applied the synthesis to trichormamide A and a diastereoisomer containing d-*allo*-Ile in position 9 instead of l-Ile. Indeed, all peptides of the laxaphycin A family have been reported with a d-*allo*-Ile^9^, except lobocyclamide A which could contain a l-*allo*-Ile, but here again the stereochemistry has not been clearly determined. It could therefore be that lobocyclamide A and trichormamide A both contain a D-*allo*-Ile at this position. The analysis of the NMR spectra of the two diastereoisomers and their comparison with the reported data did not allow structure confirmation, in part due to the lack of supply of the natural molecule.

**Figure 28:**
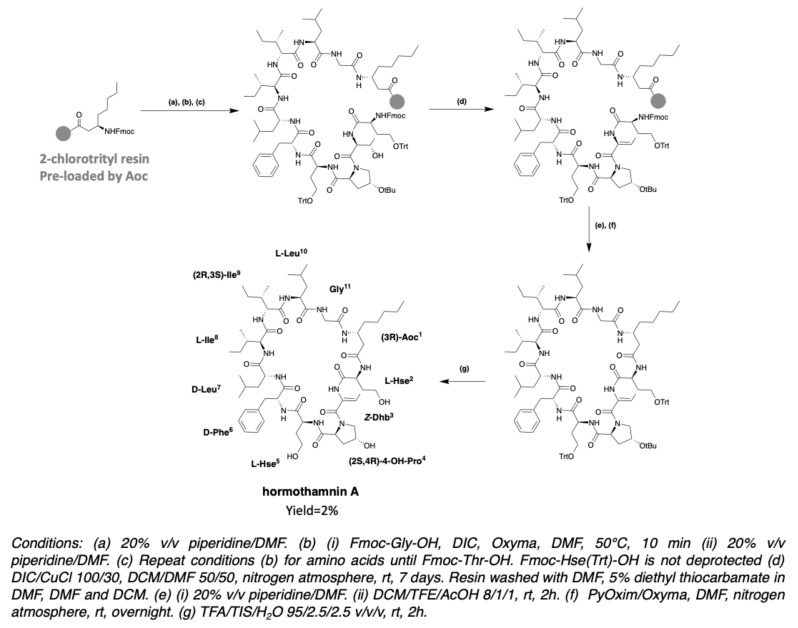
Optimized synthesis of hormothamnin A.

After obtaining trichormamide A, the same team undertook the synthesis of hormothamnin A [78]. Hormothamnin A has a *Z-*configuration dehydrobutyrine (*Z*-Dhb) in position 3. In the case of hormothamnin A synthesis, it was chosen to couple a threonine, subsequently dehydrated on the resin. The synthesis is carried out on a 2-chlorotrityl chloride resin preloaded with Aoc. Couplings are done at 50 °C with DIC/Oxyma coupling agents. The elongation of the peptide is done until the insertion of homoserine in position 2, and without deprotection of the Fmoc group of the latter. The l-threonine in position 3 introduced without side chain protection was dehydrated on resin, by the action of DIC/CuCl 100/30 in a 50/50 DCM/DMF mixture. The dehydrated peptide is then cleaved from the resin with a DCM/TFE/AcOH 8/1/1 mixture in order to keep the side chain protections. Cyclization is performed with PyOxim/Oxyma under high dilution, and then the side chain protections are removed (Figure 28). LC-MS co-injection of the peptide obtained with laxaphycin A revealed 2 distinct peaks, suggesting that the hormothamnin A (*m/z* 1196.48 for [M + H]^+^ ion) was obtained with the right Dhb configuration (*Z*-Dhb), since this is the only difference with laxaphycin A, which contains an *E*-Dhb [78].

Laxaphycin B, lyngbyacyclamide A, and trichormamide C for laxaphycin-B type peptides, and trichormamide A and hormothamnin A for laxaphycin-A type peptide, are the only peptides that have been synthesized to date. In the case of 12-residue laxaphycins, two types of synthesis have been reported, each with advantages and inconveniences. It is possible to start the synthesis by grafting a Rink Amide resin on the asparagine side chain in position 8, allowing a direct cyclization on the resin but using in excess all the amino acids, especially the non-proteinogenic ones. On the other hand, β-aminodecanoic acid can be grafted first on a chlorotrityl resin in order to spare it, but the cyclization must then be done in solution. In the case of 11-residue laxaphycins, only the second technique is possible since these compounds do not contain amino acids with side chains that can be grafted to a resin, except for scytocyclamides A and A2, and trichormamide D containing a glutamine in position 2, but the effectiveness of such a technique on the synthesis of these peptides remains to be proven.

## 4. Biosynthesis

Cyanobacteria produce a wide variety of natural bioactive products with high potential for valorization as drugs candidates. Understanding their biosynthesis would allow to consider their production methods through synthetic biology [89]. Many natural products from cyanobacteria are synthesized by nonribosomal peptide synthetase (NRPS) or polyketide synthase (PKS) and often by hybrid NRPS/PKS pathways. These large NRPS enzymatic complexes are organized in modules that activate an amino acid, modify it in certain cases before a transfer to the amino acid of the adjacent module to form the amide bond. A module contains at least one adenylation domain (A) which allows the specific recognition of an amino acid and its activation into adenylated aminoacyl, a thiolation domain (T or PCP for peptidyl carrier protein) which retains the peptide during elongation through a thioester bond, and a condensation domain (C) allowing the formation of the peptide bond. The first amino acid is activated by the A domain with ATP co-substrate in the form of an aminoacyl-adenylate (aminoacyl-AMP) and loaded on the 4’-phospho-pantethine (Ppant) side chain of the PCP domain by the action of the thiol group on the—*O*-AMP part of the activated amino acid. At this stage, the substrate can undergo modifications such as epimerization (E domain) or methylation (NMT domain) by optional secondary domains [90]. The E domain possesses a base that deprotonates the alpha position of the l-amino acid to produce an enolate. During reprotonation, an L/D mixture is obtained, but due to a strong specificity, the following C domain recognizes only the d-amino acids [91]. Different work is underway to shed light on the mechanism of action of these enzymes [92].

**Figure 29:**
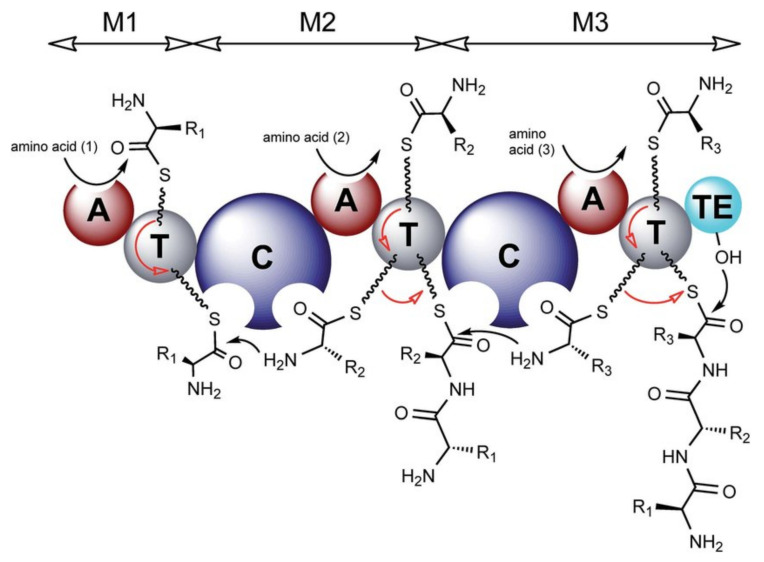
Organization of modules in NRPS synthesis [95]. A = adenylation domain, T = thiolation domain, C = condensation domain. The red arrows represent the movement of residues along the NRPS chain. This figure was taken from the review of Winn et al. [95] published under the terms of CC attribution 3.0.

The amine of the next amino acid, already anchored on its T domain, then attacks the thioester function of the previous amino acid in order to continue the elongation. At the end of the synthesis, the peptide is released by a thioesterase domain (TE) containing a serine that attacks the thioester bond between the peptide and the T domain to form a new intermediate that can either be hydrolyzed to obtain a linear peptide or undergo an intramolecular reaction to give a cyclic peptide (Figure 29). The action of the A domains is specific to the side chains of the substrates, while the action of the C domains depends on the stereochemistry of the amino acids. This high specificity ensures the synthesis of the right peptide sequence [93]. Unlike ribosomal peptide synthesis, which leads to peptides containing only natural amino acids, NRPS induce the inclusion of hundreds of non-proteinogenic residues, including d-amino acids, *N*-methylated or β-hydroxylated amino acids in peptides through the modification of natural amino acids. Hydroxy groups can be used for various modifications, such as glycosylation, and are found in many antibiotic peptides such as lysobactin or skyllamycin [90].

Many natural products are produced by hybrid NRPS/PKS pathways. Polyketide synthases (PKS) assemble small acylated blocks into polyketides via C-C bonds, and NRPS assemble amino acids into peptides via amide bonds. NRPS and PKS use a similar biosynthesis strategy with carrier proteins to attach the growing chain [94]. PKS contain an acyltransferase domain (AT), a ketosynthase domain (KS) and an acyl carrier protein domain (ACP). The AT domain recognizes an acyl unit and catalyzes its transfer to the phosphopanthein arm of the ACP to form a thioester. The KS domain first receives the acyl intermediate from the previous ACP donor via a cysteine.

**Figure 30:**
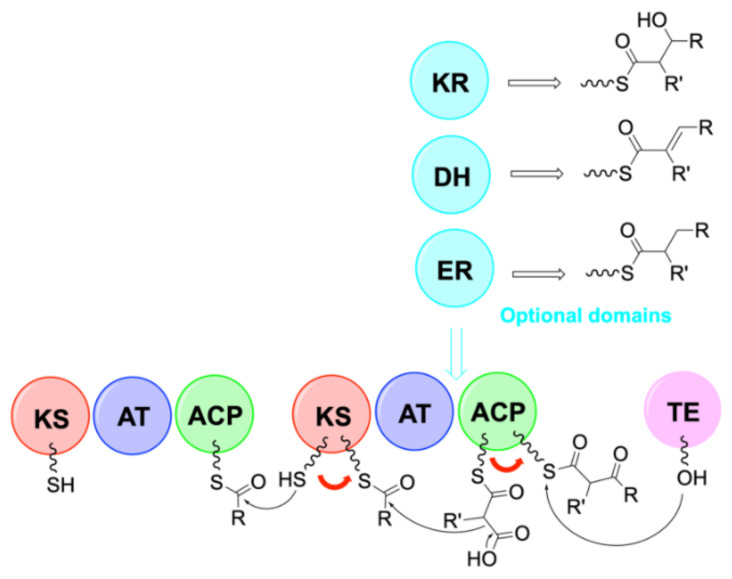
Simplified model of PKS synthesis. KS = ketosynthase domain, AT = acyltransferase domain, ACP = acyl carrier protein domain, KR = ketoreductase domain, DH = dehydratase, ER = enoyl reductase. The red arrows represent the movement of residues along the PKS chain.

The KS domain then catalyzes the decarboxylation of the growing malonyl substrate attached to the next ACP acceptor and then catalyzes a Claisen condensation between the acyl intermediate and the substrate to form a C-C bond. The resulting substrate can be treated with ketoreductase domains (KR) to form β-hydroxy groups, dehydratase (DH) to create double bonds, and enoyl reductase (ER) to form reduced methylene groups. The polyketide is then released by a thioesterase as in the case of NRPS (Figure 30). When a NRPS module is upstream of a PKS module, the last domain of the NRPS module must be recognized by the first domain of the PKS module, then condensation occurs via the KS domain. On the contrary, when a PKS module is placed before a NRPS module, the C domain of the NRPS must recognize the ACP domain of the PKS module. The combination of the NRPS and PKS systems results in products with a wide structural diversity. Wang’s team reported that one third of the PKS and NRPS gene clusters identified were hybrids [96].

### 4.1. Biosynthesis of Characteristic Amino Acids in Laxaphycins

#### 4.1.1. Biosynthesis of Hydroxy-Amino Acids

In general, the hydroxylation of amino acids can take place via three types of enzymes: FAD-dependent monooxygenases, non-heme Fe(II)-oxygenases and heme Fe(II)-oxygenases. These enzymes use different co-factors depending on the reactivity of the amino acid to be hydroxylated. This may be the iron coming either from heme in the case of monooxygenases of the cytochrome P450 family, or being in non-heme mononuclear form. Another co-factor is FAD (flavin adenine dinucleotide), which oxidizes in the presence of oxygen to form a less reactive compound, capable of hydroxylating in particular lysine amines, sufficiently nucleophilic for that. In the biosynthesis of peptides, one finds mainly P450 cytochromes or Fe(II)/α-KG-dependent non-heme monooxygenase (KG for ketoglutarate).

The biosynthesis of 3-hydroxyleucine has been reported for several peptides, most recently in the biosynthesis of scytocyclamides [38]. Heinilä et al. conducted an experiment to determine whether NRPS modules were specific for the hydroxylated amino acid or not, by supplementing the cyanobacterial culture medium with 3-hydroxyleucine. However, this experiment did not show a higher production of hydroxyleucine-containing scytocyclamide compared to the control without the addition of 3-hydroxyleucine. The two adenylation domains of the LxaI_2_ and LxaJ_1_ modules would therefore be specific for leucines and not for 3-hydroxyleucines, which was demonstrated by a comparative analysis on AntiSMASH. In the case of scytocyclamides, the hydroxylation of leucines and asparagine would therefore occur after synthesis of the complete peptide. Besides, the LxaF and LxaG modules code for enzymes of the cupin 8 family that are suspected to be involved in the β-hydroxylation of amino acids as it was described for other peptides. [97,98,99].

In the skyllamycin biosynthetic gene cluster, the monooxygenase P450 (*sky*32) allows the hydroxylation of leucine as well as tyrosine and phenylalanine [100]. This enzyme is capable of hydroxylating three different amino acids directly on the respective PCP domain.

In the same way, a P450 monooxygenase (*tem*23) showing similarities with *sky*32 (78% according to Blast searches) is able to specifically β-hydroxylates the leucine found in telomycin antibiotic [101]. The hydroxylation occurs once the complete peptide is assembled and cyclized, thus outside the NRPS assembly line, which is not the case with *sky*32. Another P450 monooxygenase (*tem*29) was found in the gene cluster, allowing the hydroxylation of proline into *trans*-3-OH-Pro. The *tem*32 gene codes for Fe(II)/2-OG-dependent oxygenase, which will be responsible for the hydroxylation of the second proline into *cis*-3-OH-Pro. These two enzymes are also supposed to act post NRPS synthesis.

Parkinson et al. combined metabologenomics and molecular networks to discover a new class of peptide, tyrobetaines, which are peptides produced by the NRPS pathway [102]. Among the recognized enzymes, TybP would code for a P450 monooxygenase, showing similarities with the enzyme coded by *tem*23 (74%). TybP would thus be responsible for the β-hydroxylation of leucine in tyrobetaine. No information is given on the mode and location of action of this enzyme.

Contrary to the case of telomycin biosynthesis where proline is hydroxylated after the complete synthesis of the peptide, peptides of the echinocandin family contain hydroxyprolines obtained before their integration into the peptide chain, in free form. GloF and HytE are 2-OG-dependent proline hydroxylases that are part of the biosynthesis of non-ribosomal peptides. They have been identified in the cluster of pneumocandin genes from *Glarea lozoyensis* (GloF) or echinocandin B from *Aspergillus pachycristatus* [103,104]. These enzymes hydroxylate free proline in positions 3 or 4, before it is loaded on an acyl carrier protein.

The origin of 4-hydroxyproline in scytoclamide A could not be determined since no enzyme allowing this type of reaction has been identified within the cluster of scytocylamide genes [38]. However, the study of heinamides biosynthesis showed that an enzyme in the gene cluster (LxaN) may be responsible for the hydroxylation of proline at position 4 [44].

Cases of hydroxylation of asparagine in free form have also been reported. In the gene cluster for the biosynthesis of occidiofungin B, of the burkholdine family, the *ocf*G gene has been determined as coding for a hydroxylase allowing the β-hydroxylation of asparagine and tyrosine [105]. Asparagine is hydroxylated in the free state and then inserted into the NRPS synthesis chain as the first amino acid of the sequence. Similarly, the lipopeptide CDA (daptomycin-like) is synthesized via NRPS, with a Fe(II)/α-KG-dependent oxygenase (AsnO hydroxylase) that catalyzes the β-hydroxylation of asparagine [98].

This type of Fe(II)/α-KG-dependent enzyme is also found in ribosomal-type synthesis. The linear polytheonamide peptide, composed of 49 residues with high structural diversity, was presumed to be synthesized by NRPS. However, it has recently been shown to be obtained by ribosomal pathway, with no less than 48 post-translational modifications [106]. Among them, the β-hydroxylation of asparagine is performed by a Fe(II)/α-KG-dependent oxidoreductase, encoded by *poy*E in the identified gene cluster.

#### 4.1.2. Biosynthesis of Dehydroamino Acids

**Figure 31:**
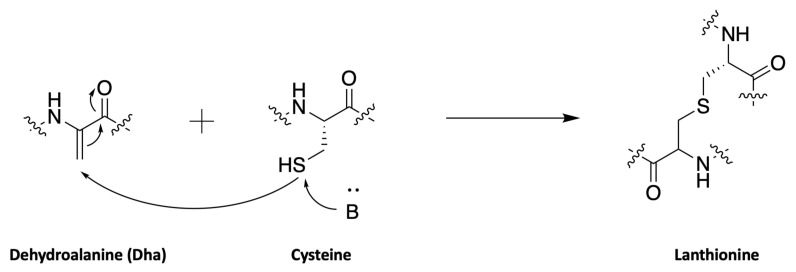
Creation of a lanthionine motif via combination of dehydroalanine and a cysteine.

The biosynthesis of dehydroamino acids is globally little known. Dehydrobutyrine is mostly described in the case of peptide synthesis by ribosomal pathways. Peptides of the large family of lantibiotics, containing in particular nisin, epidermin, or subtilin, are characterized by a lanthionine motif (Figure 31) [107]. This pattern is formed via a dehydroamino acid obtained by dehydration of the corresponding hydroxyamino acid.

In the case of class I lantibiotics, this dehydration reaction would be catalyzed by lanthionine synthetase lanB (nisB for nisin, epiB for epidermin, spaB for subtilin). Once dehydration has occurred, the addition of the thiol group of a cysteine is catalyzed by lanC, to form the lanthionine motif. In the case of class II lantibiotics, *lan*M gene is a fusion of *lan*B and *lan*C and thus codes for a bifunctional enzyme catalyzing both dehydration and formation of the lanthionine moiety [108,109].

Enzymes similar to lanthionine synthetases have been found in the thiocillin biosynthetic gene cluster. *tcl*K and *tcl*L genes are involved in the dehydration of threonines to dehydrobutyrine [110]. The mechanism of action would imply a phosphorylation of the hydroxyl group followed by its elimination [109]. Similarly, *ber*B and *ber*C from the berninamycin A gene cluster, code for dehydratases of the same type as lanthionine synthetases acting on serine and threonine [111]. Similar enzymes, homologous to LanB, are also found in the gene clusters for the biosynthesis of thiostrepton peptide [112,113] and nosiheptide peptide [114].

Dehydroamino acids are also widely found in non-ribosomally biosynthesized peptides [115,116] but their production mechanism remains very unclear [117]. Among them is the well-studied microcystin, which contains an *N*-methyl-dehydroalanine. The *N*-methyl-serine dehydration is thought to be performed by the first domain of the *Mcy*A module [118]. However, the mechanism of dehydration is still not elucidated, as this is also the case for stenothricin [119].

Concerning the NRPS synthesized puwainaphycins containing two dehydrobutyrines, a strong sequence homology (97%) of both adenylation domains (*puw*F and *puw*G) was shown without evidence for additional enzymes that could cause dehydration of threonine. Thus, the adenylation domains are suspected to be specific for dehydrobutyrine [120,121].

In the biosynthesis of scytocyclamides, the adenylation domain LxaC_2_ recognizes a threonine, then the following condensation domain LxaC_3_ would be involved in the dehydration of the threonine to dehydrobutyrine [38].

### 4.2. Biosynthesis of Laxaphycins

In the case of cyanobacteria, the first biosynthetic NRPS/PKS pathway was characterized for microcystin within the freshwater cyanobacterium *Microcystis aeruginosa* [10,122]. After this breakthrough, other peptide biosyntheses have been reported for freshwater and marine cyanobacteria. It is only very recently that the biosynthesis of laxaphycins and their derivatives has been studied [38]. For these lipopeptides, the hypothesis of a hybrid PKS/NRPS biosynthetic pathway has been anticipated [27]. In view of the structural differences between A-type and B-type laxaphycins, two different biosynthetic pathways are expected. Sivonen’s group focused on the biosynthesis of scytocyclamides from the cyanobacterium *Scytonema hofmannii* PCC 7110 [38].

**Figure 32:**
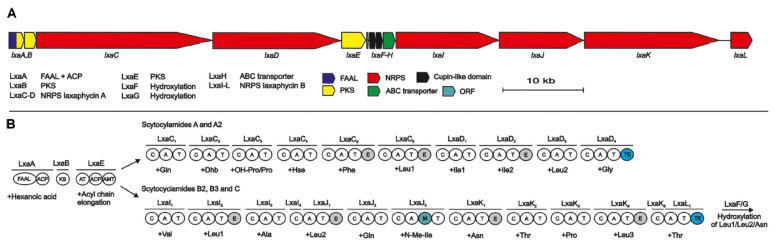
The scytocyclamide (lxa) biosynthetic gene cluster and putative biosynthetic scheme. (**A**) Organization of predicted scytocyclamide biosynthetic genes. (**B**) Proposed biosynthetic pathway of scytocyclamides. This figure was taken from the article of Heinilä et al. [38] published under the terms of CC attribution 4.0.

Analysis of the *S. hoffmannii* genome revealed 15 possible NRPS/PKS pathways but only two of them were compatible with the scytocyclamides structures. However, only one enzyme that can initiate biosynthesis was found, suggesting a common initiation step for the two A and B-types scytocyclamides, followed by a separation of the two pathways. The complete gene cluster for the biosynthesis of the two peptides is 96-kb in length and is composed of 13 open reading frames (ORFs) named ORF1 and *Lxa*A-L. The three first PKS modules LxaA-B-E that contains the fatty acyl-AMP ligase (FAAL) transform the hexanoic acid into Aoc^1^ (Figure 32) and are analogous to what is observed in the biosynthesis of other peptides such as puwainaphycin [32,120] or mycosubtilin [123]. After this step, the biosynthetic pathway splits into two distinct but parallel NRPS pathways to form either scytocyclamide A or scytocyclamide B. In the case of scytocyclamide A, the LxaC_i_-D_i_ modules continue the elongation of the peptide by adding each amino acid. LxaC_5_, LxaC_6_, and LxaD_2_ have in addition an epimerization domain accounting for the D configuration on amino acids Phe^6^, Leu^7^, and *allo*-Ile^9^, respectively. On the scytocyclamide B side, the LxaI_i_-L_i_ modules continue the elongation. The modules LxaI_2_, LxaJ_1_, LxaJ_3_, and LxaK_4_ additionally have an epimerization domain E to give an absolute configuration D on the amino acids Leu^3^, Leu^5^, Asn^8^, and Leu^11^, respectively. LxaJ_3_ also has an *N*-methylation domain to obtain *N*-methylisoleucine in position 7. At the end of the two NRPS pathways, a thioesterase TE induces a head-to-tail cyclization of the peptide and a subsequent release of the final peptide (Figure 32). LxaH is an ABC-transporter characteristic of NRPS systems that allows the transport of the peptide to its destination within the cell.

Some scytocyclamides contain hydroxylated amino acids and others do not. Experience has shown that adding 3-hydroxyleucine to the cyanobacterial culture medium does not favor the production of scytocyclamide B incorporating 3-hydroxyleucines. In accordance with the specificity analysis of the modules, this sustains that the adenylation domains LxaI_2_, LxaJ_1_, and LxaK_1_ are specific for leucines and asparagine but not for their hydroxylated counterparts. Furthermore, these amino acids are epimerized to the d-configuration within the same modules. Thus, hydroxylation would occur after the incorporation and epimerization of the amino acids and would be catalyzed by enzymes of the cupin 8 family encoded in the LxaF-G modules [124], suggesting that the hydroxylation enzymes are specific for d-amino acids, or that epimerases play a role in these hydroxylations. Concerning the obtention of 4-hydroxyproline in scytocyclamide A, no hypothesis has been put forward since no enzyme from the gene cluster has been detected as being suitable for such synthesis. The LxaC_2_ module would contain a threonine-specific A domain, and it would be the C domain of the following LxaC_3_ module that would induce dehydration of threonine to dehydrobutyrine, as already postulated in the biosynthesis of other peptides such as microcystin [118] or nodularin [115]. Similarities appeared when comparing the condensation domains observed in these peptides with the C domain of the LxaC_3_ module. Finally, LxaC_4_ module is specific of Hse.

**Figure 33:**
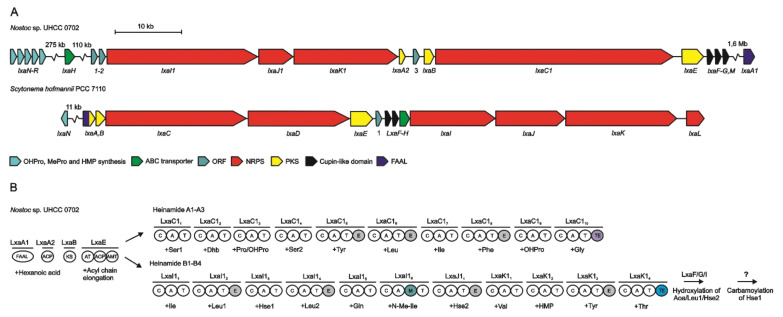
The laxaphycin (lxa) biosynthetic gene clusters and putative biosynthetic schemes in *Nostoc* sp. UHCC 0702 and *S. Hofmannii* PCC 7110. (**A**) Organization of predicted heinamide and scytocyclamide biosynthetic genes. (**B**) Proposed biosynthetic pathway of heinamides and scytocyclamides. This figure was taken from the article of Heinilä et al. [44] published under the terms of CC attribution 3.0.

In 2021, Heinilä et al. reported the biosynthetic gene cluster involved in the biosynthesis of heinamides [44]. The 93-kb complete gene cluster is composed of 13 open reading frames (ORFs) named ORF_1_, ORF_2_, *lxa*I_1_-K_1_, *lxa*A2, ORF_3_, *lxa*B, *lxa*C1, *lxa*E-G, and *lxa*M (Figure 33).

As it is the case for scytocyclamides, the Aoc^1^ is the first residue synthesized. However, no LxaA-like enzyme was found in the heinamide biosynthetic gene cluster. A single ACP domain LxaA2 was found with 41% similarity to LxaA and a single FAAL LxaA1 was designated as possibly involved in the initiation of biosynthesis but is located outside the gene cluster. This separation has already been described in the case of puwainaphycins [121]. The LxaB ketosynthase completed by an acyltransferase, an ACP domain, and an aminotransferase in the LxaE module constitute the set of enzymes allowing the Aoc^1^ production.

The LxaC1_i_ modules continue elongation for A-type heinamides while LxaI1_i_-K1_i_ are involved in the biosynthesis of B-type heinamides. Furthermore, epimerization domains are present on modules corresponding to the d-amino acids found in heinamides, as well as a methylation domain for *N*-methylisoleucine addition.

The enzymes LxaF-G and LxaM were identified as cupin-like domain proteins and would be involved in the hydroxylation of Aoc^1^, Leu^3^, and Hse^8^. The adenylation domains corresponding to Hle^3^ and Leu^5^ being identical, they would be specific to leucine and the hydroxylation of Leu^3^ would occur later, either while it is bound to the PCP or after the release of the peptide. It is noteworthy that for the different B-type laxaphycins, position 3 is always hydroxylated, which is not the case for position 5. The LxaI1_3_ and LxaJ1_1_ adenylation domains corresponding to Hse and to the carbamoylated homoserine (cHse) are identical, suggesting that both domains are Hse-specific, and that carbamoylation occurs later. Position 8 would therefore correspond to a Hse hydroxylated at position 3, rather than a Thr hydroxylated at position 4, as described in the case of lobocyclamides [30]. Indeed, in other laxaphycins containing a 3-hydroxyasparagine (Hasn), the hydroxylation is done on carbon 3. Lastly, it is not yet known at what point carbamoylation of Hse occurs. Three putative carbamoyltransferases were found in the genome, but none could be assigned to the gene cluster.

As in the case of scytocyclamides, the LxaC1_3_ condensation domain handles the dehydration of threonine to dehydrobutyrine, with threonine recognized by the preceding LxaC1_2_ adenylation domain.

The LxaO-P-Q enzymes are described as an l-Leu 5-hydroxylase, a zinc-binding dehydrogenase, and a pyrroline-5-carboxylate reductase, respectively. These enzymes are encoded by genes similar to those found in other cyanobacteria and allowing the production of 4-Me-Pro [125], but these enzymes were found outside the gene cluster. LxaN hydroxylates l-Pro to 4-OH-Pro in the case of type A heinamides. While this enzyme was not identified when scytocyclamide biosynthesis was studied, a 93% LxaN-like enzyme was found in the scytocyclamide gene cluster, also hydroxylating l-Pro for type A scytocyclamides. LxaR, on the other hand, would participate in the production of 3-OH-4-Me-Pro for type B heinamides, but does not directly hydroxylate 4-Me-Pro. Indeed, the addition of 4-Me-Pro to the medium does not increase the amount of peptide containing 3-OH-4-Me-Pro, suggesting that 4-Me-Pro is not an intermediate in 3-OH-4-Me-Pro synthesis. LxaR would be assisted by LxaO-P-Q but the direction of action of the four enzymes is not defined.

Although the gene clusters for scytocyclamide and heinamide biosyntheses are very similar, there are several differences in their organization (Figure 33A). The biosynthesis of heinamides complements the information already described for the biosynthesis of scytocyclamides, these two families of laxaphycins being the only ones for which the biosynthesis has been described to date.

The discovery of acyclolaxaphycins raises doubts about their origin. It was first postulated that these linear peptides could be by-products of an incomplete biosynthesis, or products of enzymatic degradations [27,32]. In the case of laxaphycin A, the cyclization would therefore be between Aoc^1^ and Gly^11^ (Figure 2), and in the case of laxaphycin B, between Ala^4^ and 3-hydroxyleucine^3^ (Figure 3). According to the PKS/NRPS architecture proposed by Heinilä et al., with the start of biosynthesis by Aoc^1^ (Figure 32), it is possible that acyclolaxaphycins A are an unfinished by-product, having stopped at Leu^10^ or d-*allo*-Ile^9^. In contrast, for acyclolaxaphycins B and B3, the opening is described in the middle of the NRPS gene sequence, suggesting instead that these peptides were obtained by enzymatic degradations. Furthermore, if cyclization were to take place between the amine of Ala^4^ and the carboxylic acid of 3-hydroxyleucine^3^, it would also not be possible to obtain [des-(Ala^4^-Hle^5^)]acyclolaxaphycin B and [des-(Ala^4^-Hle^5^)]acyclolaxaphycin B3 as immature products of the biosynthesis.

## 5. In Situ and Ex Situ Biological Activities

Cyanobacteria biosynthesize various secondary metabolites suspected to be involved in UV protection, feeding deterrence or allelopathy [126]. The wide range of biological properties of these metabolites made them attractive compounds for the pharmaceutical, cosmetic, agricultural, and energy domains. In this section, we will summarize the studies that have been undertaken on the biological activity of laxaphycins and their derivatives (Tables S1 and S2). We will also discuss their impact on the consumption preferences of different herbivores.

### 5.1. Biological Properties

#### Laxaphycins

Most of the laxaphycin derivatives were tested either for their antimicrobial activities or their cytotoxicities (Tables S1 and S2).

Generally speaking, A-type laxaphycins have moderated biological activities, except hormothamnin A that shows IC50 in the submicromolar range on different cancer cell lines (Table S1) [19,21,22,25,29,40,41]. In comparison, hormothamnin A has an IC50 of 0.72 μM on HCT-116 cells while laxaphycin A, differing only from hormothamnin A by the Dhb configuration, has an IC50 of 23 μM [25,29]. Nevertheless, A-type laxaphycins were further studied and compared with their acyclic analogs. On SHSY5Y neuroblastoma cells, depending on the different type A laxaphycins, a change in the electrical potential of the mitochondrial membrane accompanied by a change in the level of reactive oxygen species (ROS) was observed, suggesting an autophagy phenomenon [27].

B-type laxaphycins are more active and exhibited anticancer activity on various cell lines with IC50 lower than 2 µM but their antimicrobial activity is moderate (Table S2) [19,21,22,25,26,39,40,41].

Interestingly, type A laxaphycins potentiate the activity of type B laxaphycins when the two compounds are combined. This phenomenon was documented for almost all the cyanobacteria for which the two compound types were co-isolated albeit explanation about the mechanism of this synergism remains poorly understood [19,21,30,38,44]. The synergistic activity of laxaphycins and their derivatives would be linked to their shared biosynthesis mode. Indeed, this would suggest simultaneous regulation and expression of synergistic products to interact with the same target via different mechanisms. Such a phenomenon of co-regulation of distinct groups of genes has already been observed with the synergistic antibiotics griseoviridin and viridogrisein in *Streptomyces griseoviridis* [38,127].

Due to their higher activity, B-type laxaphycins were more studied to elucidate their mechanism of action. The intracellular effect of laxaphycins A and B was studied using videomicrofluorometry with multiple labelling. Laxaphycin B at 2 μM fully blocks the growth of CEM cells and polyploid cells are observed after the treatment [128]. Furthermore, B-type laxaphycins are active on both parent drug-sensitive human leukemic lymphoblasts (CCRF-CEM-WT) and related sublines that express drug resistance (CEM-VLB_100_, CEM/VM1), with IC50s ranging from 1.02 to 4.15 μM [22]. Laxaphycin B has antiproliferative activity on several solid cancer cell lines non-specifically and exerts a comparable cytotoxicity on normal human fibroblasts or immortalized murine L-929 cells (Table S2) [22].

B type laxaphycins have also been studied more precisely for their activity on neuroblastoma SHSY5Y cells [33]. As expected, laxaphycins B and B3 are cytotoxic against these cells, with IC50s between 0.3 and 1.8 μM (according to MTT or LDH tests) for laxaphycin B and between 0.15 and 0.8 μM for laxaphycin B3. These two cyclic peptides depolarize the mitochondrial membrane and reduce ROS and ATP levels. On the contrary, acyclolaxaphycin B and B3 as well as [des-(Ala^4^-Hle^5^)]acyclolaxaphycin B and B3 do not show cytotoxicity at all concentrations tested but affect ROS and ATP levels. These acyclic laxaphycins induce changes in the electrical potential of the mitochondrial membrane, except [des-(Ala^4^-Hle^5^)]acyclolaxaphycin B3. The addition of laxaphycins B and B3 resulted in a decrease of living cells and an increase of 50% and 46% respectively of apoptotic cells. In contrast, acyclic laxaphycins showed no effect on cell viability. Caspase 3 is an enzyme targeting apoptosis substrates and generating a series of events leading to cell death. The activity of this caspase is increased by the addition of laxaphycins B and B3 and not by the addition of acyclic laxaphycins, reinforcing the idea that cyclic laxaphycins B are involved in the apoptosis of neuroblastoma cells. Since acyclic laxaphycins show an effect on the potential of the mitochondrial membrane, their involvement in cell autophagy was evaluated. Neuroblastoma cells were treated with different laxaphycins and the expression of different proteins involved in autophagy (rapamycin, compound C AMPK inhibitor, p70 S6 kinase, LC3, p62) was determined. Acyclic laxaphycins B induce an increase in AMPK activation, thus activating autophagy, with [des-(Ala^4^-Hle^5^)]acyclolaxaphycin B3 being the most impacting. Cyclic laxaphycins have no impact on mTOR activity, while acyclic laxaphycin B reduces its activation. Laxaphycin B3 and its two acyclic derivatives show a decrease in p70 S6 phosphorylation, involved in both autophagy and apoptosis activation, suggesting that laxaphycin B3 has a different mechanism of action than laxaphycin B. The hydroxy group on proline in position 10 could therefore have an impact on the inhibition of this enzyme. In addition, the LC3-II/I ratio is increased by the addition of acyclic laxaphycins to the cells. The expression of p62 is decreased by the addition of these acyclic laxaphycins. Treatment of neuroblastoma cells with cyclic laxaphycins would therefore act on their apoptosis, while acyclolaxaphycins would be implicated in their autophagy. Immunofluorescence study of HeLa cells with antibodies raised against alpha-tubulin showed that scytocyclamide B causes depolymerization of the protein [9,37].

Different species of algae and cyanobacteria are used as food supplements. Many potential beneficial uses have been associated with extracts and compounds of cyanobacteria and algae, including antioxidant, immunomodulatory, antimicrobial, or antitumor activities [129]. Dussault et al. evaluated the antibacterial activity of laxaphycins A, B, and B3 against five foodborne pathogens, the Gram-positive strains *Listeria monocytogenes*, *Staphylococcus aureus,* and *Bacillus cereus*, and the Gram-negative strains *Escherichia coli* and *Salmonella enterica* Typhimurium. Laxaphycins are not active on Gram-negative strains, a result that can be explained by the hydrophobicity of the structure of these peptides. Indeed, the membrane of Gram-negative strains contains lipopolysaccharides that create a hydrophilic layer to prevent the entry of hydrophobic compounds into the cell. Laxaphycins show no or moderate activity on Gram-positive strains (Tables S1 and S2).

### 5.2. Feeding Deterrence as an Example of Ecological Role

Secondary metabolites play an important role in the interactions between different organisms within the trophic chain [34,130]. Some compounds are toxic and have been selected by cyanobacteria as an effective defense strategy against herbivorous predators. But these predators can also develop strategies of resistance to repellent, antiappetant, or toxic compounds, allowing them to feed and hide from their own predators. Laxaphycins and their derivatives could show toxicity on certain organisms. It is therefore highly probable that the associated cyanobacteria synthesize them in order to protect themselves from herbivores, competitors, or fouling organisms. However, the ecological role of laxaphycins remains unclear [9]. Laxaphycin A, hormothamin A, and lyngbyacyclamides are non-toxic on brine shrimp *Artemia*, while laxaphycin B is lethal [28,29,39]. Hormothamnin A is, on the other hand, toxic on the goldfish *Carassius carassius* with an LD50 of approximately 5 μg/mL [29]. Scytocyclamides are toxic against the crustacean *Thamnocephalus platyurus* after only two hours, with an LD50 of about 12 μmol for scytocyclamide A and 4 μmol for scytocyclamide B [37]. Given the different responses obtained with these peptides on different organisms, the use of all laxaphycin-type peptides as chemical defenses cannot be confirmed.

It has been shown that the cyanobacterium *H. enteromorphoides* is chemically defended against most of these predators [131]. Out of eleven cyanobacteria, algae, and sea grasses tested, *H. enteromorphoides* was one of the few to repel the sea hare *Dolabella auricularia*, suggesting a chemical repulsion phenomenon around this cyanobacterium. This phenomenon of repulsion was also observed on various fish. Indeed, different fractions extracted from this cyanobacterium have been evaluated for their ability to repel predators. The fraction containing cyclic peptides, including laxaphycin A, was able to avoid predation, whereas the fractions containing no cyclic peptides did not. However, not all predators respond in the same way to the mixture of peptides. For example, pufferfish *C. solandri* is weakly impacted by the chemical environment of the cyanobacterium while sea urchin *D. savignyi* is completely repelled by the mixture of peptides. Biosynthesizing several peptides could therefore be a way for the cyanobacterium to repel a wider range of predators, with the hypothesis that each of them has a different sensitivity to each peptide. Moreover, these peptides may have a synergistic effect on each other. However, there is no evidence that the synergism observed in toxicity tests is also observed on the palatability of herbivores. In fact, in this study, laxaphycin A was as effective, or even slightly more effective, in repelling *S. schlegeli* parrotfish as was the mixture of peptides at the same concentration [131].

Although a few studies have been done on the impact of secondary cyanobacterial metabolites on predation [130,132,133,134], very little research has involved laxaphycins and their derivatives. Among these studies, many have shown that the sea hare *Stylocheilus striatus* was not repelled by certain secondary cyanobacterial metabolites. Compared to four other generalist herbivores, *S. striatus* is the only one not repelled by the cyclic peptide pitipeptolide A biosynthesized by *Lyngbya majuscula* [135]. Considered as a specialist of *L. majuscula*, it could have adapted to its chemical environment. However, this mollusk was also found on *A. torulosa*, known to synthesize laxaphycins in particular [34]. By comparing by LC-MS the chemical fingerprints of *A. torulosa* and *S. striatus*, Louis Bornancin discovered that laxaphycin A was sequestered within the digestive gland of the mollusk, while laxaphycins B and B3 were present in the cyanobacterium but no more within the mollusk, presenting new acyclolaxaphycins previously described [33,34], suggesting a degradation of cyclic laxaphycin-B type peptides [32]. *S. striatus* would thus be involved in a biodegradation process of laxaphycin B, allowing it to detoxify this cyclic peptide [43,88]. The mollusk could have adapted to this unattractive cyanobacterium to hide from its main predators. Indeed, Louis Bornancin observed in the field that *Gymnodoris ceylonica*, specialist predator of *S. striatus*, and *Thalamita coerulipes*, generalist predator of some herbivores, were present on *L. majuscula* but absent from *A. torulosa*. Secondary metabolites of *A. torulosa* could then repel these two predators [34]. In order to understand the adaptation mechanism of *Stylocheilus striatus*, Darcel et al. further investigated the biodegradation of B-type laxaphycins within the mollusk digestive gland [88]. By synthesizing several analogues of laxaphycin B, they found that the mollusk digestive gland contained an enzyme capable of cleaving laxaphycin B and its analogues at the *C*-terminal amino acids at positions 3 and 5. They also demonstrated that the enzyme was a d-peptidase, capable of linearizing cyclic peptides through a two-step cleavage. The few d-peptidases known to date are of bacterial origin [136]. *Stylocheilus striatus* could therefore have either recovered the enzyme from its environment or produced it itself, perhaps as a protective measure against the toxicity of these peptides.

## 6. Conclusions

To date, the family of laxaphycins includes 16 A type laxaphycins and 27 B type laxaphycins, mostly cyclic peptides. Acyclolaxaphycins are either derived from incomplete biosynthesis or are enzymatic degradation products [27,32,38]. Laxaphycins and their derivatives have a wide structural diversity, including many non-proteinogenic amino acids and d-configured amino acids. However, their structures remain very close, with changes in amino acids keeping the same absolute configuration and being mostly isosteric. Since these amino acids have relatively close structures and the patterns can be repeated within the peptide, researchers have difficulties in determining the structures and stereochemistries with certainty. Despite using increasingly precise techniques such as NMR or mass spectrometry, errors in attribution are often observed for natural substances [24]. The chemical synthesis of these peptides is the best way to confirm a proposed structure, since it allows the comparison of the synthetic peptide with the natural molecule. The total synthesis of laxaphycin B was the first to be described [23]. However, the synthesis of this type of peptide is complex and requires optimizations. In view of the specific structure of these peptides, the possible synthesis methods are limited, and it is necessary to synthesize beforehand the various non-proteinogenic and non-commercial amino acids. Some syntheses have been described, such as that of trichormamides A and C [42,43]. It is possible to consider the application of these synthesis techniques to other peptides of the same family in order to confirm their structure later on. The structural similarities of these peptides suggest that they are biosynthesized in the same way. Since laxaphycins were isolated from various cyanobacteria harvested at different localities, their origin could be the result of horizontal gene transfer between different cyanobacteria [31,32]. The biosynthesis of these peptides has been very little studied, since the only studies identified to date are that of the biosynthesis of scytocyclamides and heinamides, published very recently [38,44]. This study suggests that A type and B type scytocyclamides and heinamides share common PKS modules to initiate their biosynthesis with β-aminooctanoic acid. Subsequently, two distinct NRPS pathways, matching with the structure of the two peptide types, act in parallel to elongate the rest of the sequence to obtain either scytocyclamides A or scytocyclamides B. This result is interesting because the majority of NRPS pathways generally follow the collinearity rule, even if some exceptions are reported in the literature [121,137]. Furthermore, it is not common to have genes involved in the biosynthesis of an NRPS/PKS product that are located outside the gene cluster, although there are examples of gene cluster crossovers at different locations in the genome [138]. Most of the genes involved in the biosynthesis of laxaphycins have been described, but there are still question marks as to the origin of some amino acids, such as dehydrobutyrine. Although some enzymes capable of hydroxylating particular amino acids are known, their mode and location of action are not very detailed. β-hydroxylation can take place on the free amino acid upstream of elongation, directly on the PCP domain during elongation or post-synthesis.

The great diversity of amino acids making up laxaphycins can confer many biological activities to them. The vast majority of these peptides have antibacterial and antifungal activities, and can even be cytotoxic on many cancer cell lines [22,27,29,33,39,40,41]. However, cytotoxic peptides appear to be non-specific between normal and cancerous cell lines [22]. A type laxaphycins, showing little or no cytotoxic activity on their own, act in synergy with the more toxic B type laxaphycins. A Type laxaphycins potentiate the action of B type laxaphycins both on cells and on the growth of bacteria and fungi. However, it seems that these two types of peptides act by different biological pathways. Their toxicity acting as a chemical shield could also help repel predators of the cyanobacteria. Since all peptides have different activities, one might wonder why cyanobacteria synthesize so many peptides, rather than simply synthesizing the peptides that repel predators the most. It has been shown that each predator has a different sensitivity to each molecule, so it would be more interesting for cyanobacteria to synthesize a wide range of peptides to repel more predators [131]. The synergy observed with laxaphycins A and B for cytotoxicity was not found in cases of predation. The diversity of peptides synthesized by cyanobacteria is also related to multiple specificities of adenylation domains in the NRPS chain, allowing the introduction of isosteric amino acids, for example [139].

In the 30 years since their first discovery, the family of laxaphycins has expanded as researchers have become more knowledgeable about them. We are now able to characterize and synthesize these peptides. Their complex structures are of great interest as to their origin, and also their ecological and biological roles. While some questions remain unanswered, studying the ecological role of laxaphycin has already opened new possibilities such as the discovery of an unexpected d-peptidase involved in their biotransformation [88].

## Figures and Tables

**Table 1:** Amino acid sequence and molecular weight of laxaphycin A analogues.

% AA Variation vs LaxA		1	2	3	4	5	6	7	8	9	10	11	Exact Mass
54.5%	Heinamide A3		Ser		Pro	Ser	(2R)Tyr			(2R)Phe	Pro		1186.65122
	Heinamide A2		Ser		Pro	Ser	(2R)Tyr			(2R)Phe	(2S,4R)Hyp		1202.64614
45.5%	Heinamide A1		Ser			Ser	(2R)Tyr			(2R)Phe	(2S,4R)Hyp		1218.64105
18.2% or 27.3%	Lobocyclamide A		Ser				(2R)Tyr			(2R,3S)Ile or (2S,3R)Ile			1197.70091
18.2%	Scytocyclamide A2		Gln		Pro								1206.73763
	[Des-(Leu^10^-Gly^11^)]AcyclolaxA												1043,62669
9.1%	Scytocyclamide A		Gln										1222.73255
	Hormothamnin A			Z-Dhb									1195.72165
	[D-Val^9^]LaxA									(2R)Val			1181.70599
	LaxA2								Val				1181.70599
	[Des-Gly^11^]AcyclolaxA												1156,71075
	**AcyclolaxA**												1213,73221
	**LaxA**	**Aoc**	**Hse**	**E-Dhb**	**(2S,4R)Hyp**	**Hse**	**(2R)Phe**	**(2R)Leu**	**Ile**	**(2R,3S)Ile**	**Leu**	**Gly**	1195.72165
9.1%	LaxE	Ade											1223.75295
63.6%	Trichormamide D	Ade	Gln		Pro	Ser	(2R)Tyr		Val	(2R)Phe			1256.71989
63.6% or 72.7%	Trichormamide A	Ade	Ser	Ser	Pro	Ser	(2R)Tyr			(2R,3S)Ile or Ile	Pro		1183.68526

Columns highlighted in orange indicate amino acids being the same in all analogues compared to the laxaphycin A sequence. Columns highlighted in blue show partially conserved amino acids in the analogues relative to the laxaphycin A sequence. When amino acids are different between laxaphycin A and the analogues, the replaced amino acids are reported in a white box. Empty white boxes indicate the absence of the corresponding amino acids compared to laxaphycin A. The grey boxes represent the amino acids for which there is still a doubt about the stereochemistry of the amino acid. Aoc: 3-aminooctanoic acid, Ade: 3-aminodecanoic acid, Hse: homoserine, Dhb: dehydrobutyrine, Hyp: hydroxyproline.

**Table 2:** Amino acid sequence and molecular weight of laxaphycin B analogues.

% AA Variation vs LaxB		1	2	3	4	5	6	7	8	9	10	11	12	Exact Mass
41.7%	LaxB6		Ile		Val	(2R)Leu			(2R)Asn			(2R)Tyr		1454.87486
33.3%	LaxB5		Ile		Val				(2R)Asn			(2R)Tyr		1470.86977
	Trichormamide B		Ile		Hse				(2R)Ser			(2R)Tyr		1445.83814
	Lyngbyacyclamide B				Hse	(2R)Leu					(2S,4R)Hyp	(2R)Phe		1458.83338
25%	Lyngbyacyclamide A				Hse	(2R)Leu						(2R)Phe		1442.83847
	[Des-(Ala^4^-Hle^5^)]AcyclolaxB1211						Δ							1211.70131
	[Des-(Ala^4^-Hle^5^)]AcyclolaxB3										(2S,4R)Hyp			1228.72786
16.7%	Lobocyclamide B								3-OHHse*		(2S,4R)Hyp			1397.83814
	LaxB4				Hse						(2S,4R)Hyp			1440.84395
	[Des-(Ala^4^-Hle^5^)]AcyclolaxB1195						Δ							1195.70639
	[Des-(Ala^4^-Hle^5^)]AcyclolaxB													1212.73294
8.3%	AcyclolaxB3										(2S,4R)Hyp			1428.84395
	LaxB3										(2S,4R)Hyp			1410.83339
	LaxB2					(2R)Leu								1378.84355
	Trichormamide C								(2R)Asn					1378.84355
	AcyclolaxB													1412.84904
	**LaxB**	**Ade**	**Val**	**(2R,3S)Hle**	**Ala**	**(2R,3S)** **Hle**	**Gln**	**(N)-MeIle**	**(2R,3R)** **Hasn**	**Thr**	**Pro**	**(2R)Leu**	**Thr**	1394.83847
8.3%	LaxD or Scytocyclamide B	Aoc												1366.80717
16.7%	Scytocyclamide B2	Aoc							(2R)Asn					1350.81226
	Scytocyclamide C	Aoc				(2R)Leu								1350.81226
25%	Scytocyclamide B3	Aoc				(2R)Leu			(2R)Asn					1334.81734
	Lobocyclamide C	Aoc							3-OHHse*		(2S,4R)Hyp			1369.80684
50.0%	Heinamide B3	Aoc	Ile		O-carbamoyl-Hse	(2R)Leu			3-OHHse	Val		(2R)Tyr		1472.84903
58.3%	Heinamide B2	5-OH-Aoc	Ile		O-carbamoyl-Hse	(2R)Leu			3-OHHse	Val		(2R)Tyr		1488.84395
	Heinamide B4	Aoc	Ile		O-carbamoyl-Hse	(2R)Leu			3-OHHse	Val	3-OH-4-MePro	(2R)Tyr		1502.85960
66.7%	Heinamide B1	5-OH-Aoc	Ile		O-carbamoyl-Hse	(2R)Leu			3-OHHse	Val	3-OH-4-MePro	(2R)Tyr		1518.85451
	Heinamide B5	5-OH-Aoc	Ile		O-carbamoyl-Hse	(2R)Leu			3-OHHse	Val	4-MePro	(2R)Tyr		1502.85960

Columns highlighted in orange indicate amino acids being the same in all analogues compared to the laxaphycin B sequence. Δ sign represents cyclic glutamine and is not considered as a modification in the sequence as the cyclization occurs after the deletion of Ala^4^ and Hle^5^. Columns highlighted in blue show partially conserved amino acids in the analogues relative to the laxaphycin B sequence. When amino acids are different between laxaphycin B and the analogues, the replaced amino acids are reported in a white box. Empty white boxes indicate the absence of the corresponding amino acids compared to laxaphycin B. *reported as hydroxythreonine. Aoc: 3-aminooctanoic acid, Ade: 3-aminodecanoic acid, Hle: hydroxyleucine, Hse: homoserine, (*N*)-MeIle: *N*-methylisoleucine, Hasn: hydroxyasparagine, Hyp: hydroxyproline.

## Supplementary Materials

The following are available online at https://www.mdpi.com/article/10.3390/md19090473/s1, Table S1: Summary of the biological activity of laxaphycins A and their derivatives, Table S2: Summary of the biological activity of laxaphycins B and their derivatives. Figure S1: Similarity mass network linking the laxaphycin A analogues. Dark yellow arrows rep-resent one or more amino acid changes, including loss of hydroxy groups. The light-yellow arrows indicate a ring opening or loss of amino acids without modifications. The white arrow to hor-mothamnin A shows that there are no modifications or loss of amino acids but simply a change in stereochemistry. Lax: laxaphycin, Hor: hormothamnin, Lob: lobocyclamide, Scy: scytocyclamide, Acyclolax: acyclolaxaphycin, Tc: trichormamide. Figure S2: Similarity mass network linking the laxaphycin B analogues. Dark yellow arrows represent one or more amino acid changes, including loss or gain of hydroxy groups. The light-yellow arrows indicate a ring opening or loss of amino acids without modifications, including the cyclization of glutamine to pyroglutamate. Lax: lax-aphycin, Lob: lobocyclamide, Lyn: lyngbyacyclamide, Scy: scytocyclamide, Acyclolax: acyclolax-aphycin, Tc: trichormamide.
