# Supplementary material for: Thirtieth Anniversary of the Discovery of Laxaphycins. Intriguing Peptides Keeping a Part of Their Mystery
Source: Mar Drugs. 2021 Aug 24;19(9):473. doi: 10.3390/md19090473 (PMC8471579; doi:10.3390/md19090473)
Supplement: Supplementary file 1 [file marinedrugs-19-00473-s001.zip › marinedrugs-1339562-supplementary.pdf]

Supporting Information

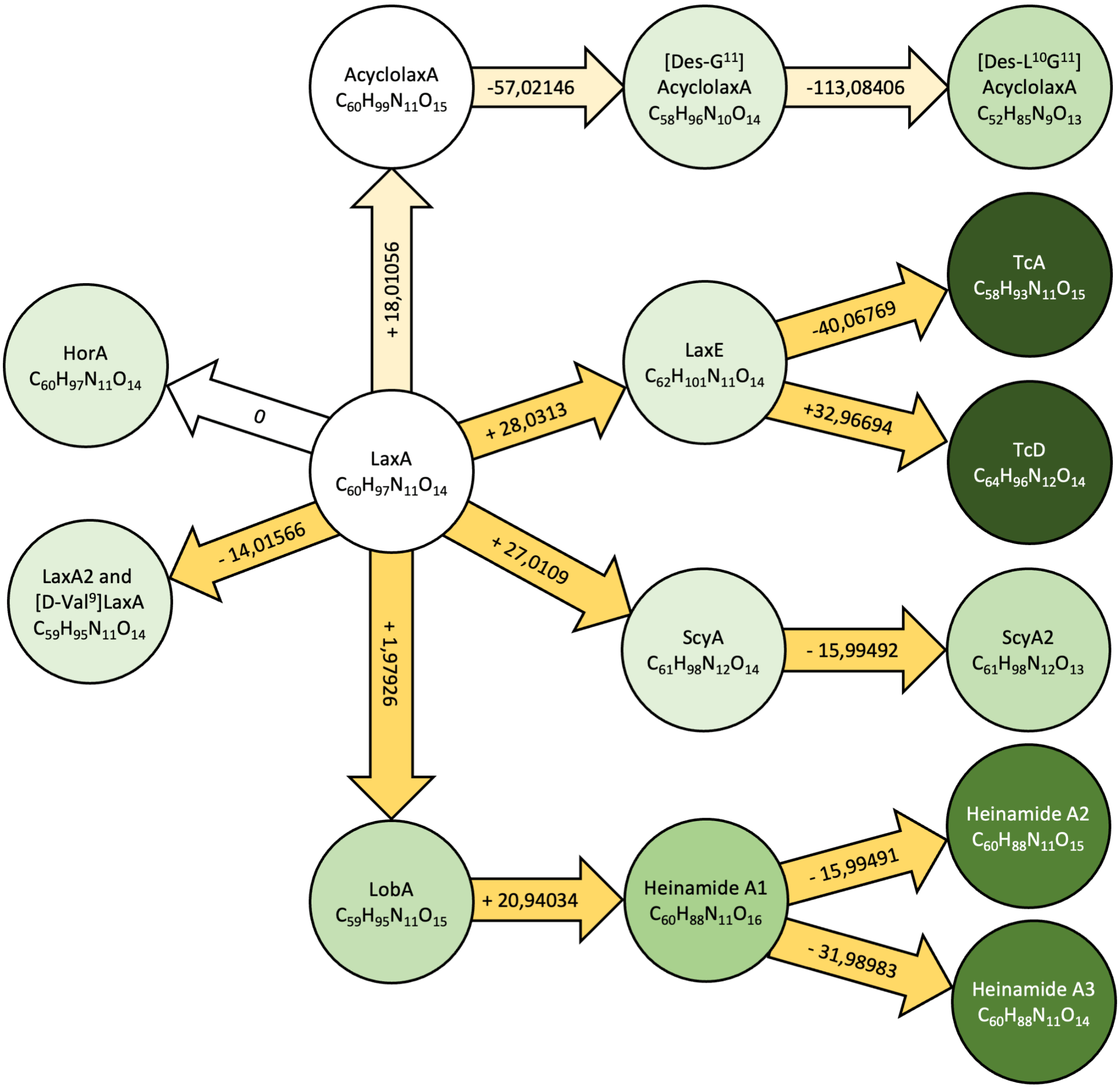

Figure S1. Similarity mass network linking the laxaphycin A analogues. Dark yellow arrows represent one or more amino acid changes, including loss of hydroxy groups. The light-yellow arrows indicate a ring opening or loss of amino acids without modifications. The white arrow to hormothamnin A shows that there are no modifications or loss of amino acids but simply a change in stereochemistry. Lax: laxaphycin, Hor: hormothamnin, Lob: lobocyclamide, Scy: scytocyclamide, Acyclolax: acyclolaxaphycin, Tc: trichormamide.

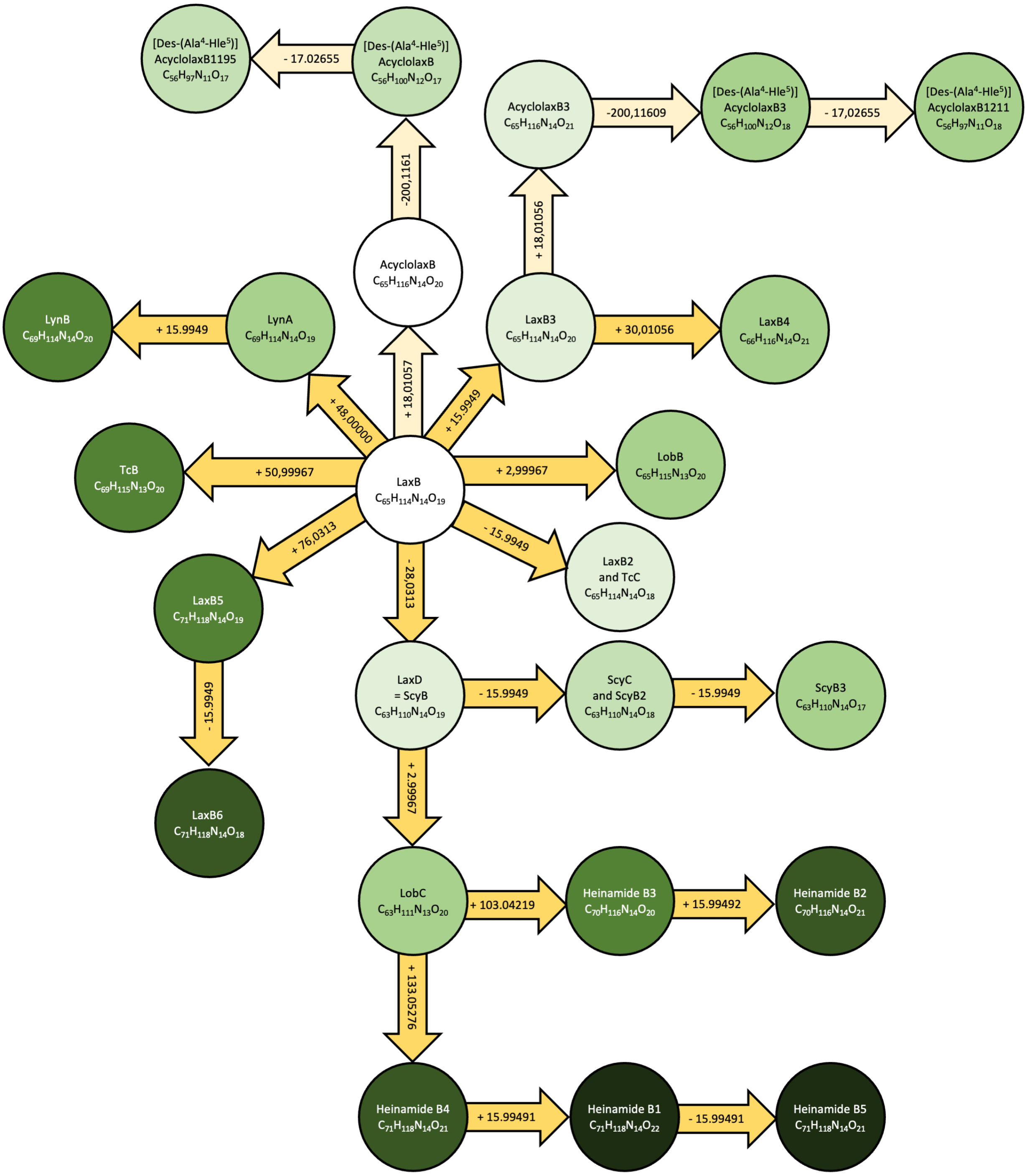

Figure S2. Similarity mass network linking the laxaphycin B analogues. Dark yellow arrows represent one or more amino acid changes, including loss or gain of hydroxy groups. The light-yellow arrows indicate a ring opening or loss of amino acids without modifications, including the cyclization of glutamine to pyroglutamate. Lax: laxaphycin, Lob: lobocyclamide, Lyn: lyngbyacyclamide, Scy: scytocyclamide, Acyclolax: acyclolaxaphycin, Tc: trichormamide.

Table S1. Summary of the biological activity of laxaphycins A and their derivatives. For the purpose of clarity, the intracellular properties of the peptides have not been reported. Dose, Activity, Target. NA=Not active.

Laxaphycin A family (11 residues)	Cytotoxicity	Antimicrobial activity	Antifungal activity	Toxicity on animals	Ref.
**Laxaphycin A**	10 µg/mL, NA on KB and LoVo cells. 20 µg/mL, NA on CCRF-CEM-WT, CEM/VLB and CEM/VM1 cells. NA on A549, MCF7, PA1, PC3, DLD1, M4Beu, HCT116, SHSY5Y solid cancer lines.	NA on *E. coli* and *S. enterica* Typhimurium. MIC 125 µg/mL on *S. aureus*. MIC 250 µg/mL on *B. cereus*, *L. monocytogenes*.	128 µg/mL, NA on *A. oryzae*, *C. albicans*, *P. notatum*, *S. cerevisiae* and *T. mentagrophytes.* Synergism with laxaphycin B on all strains.	200 µg/mL NA on *A. salina.*	[1–8]
**Laxaphycin A2**	NA on HCT116. IC50 0.6 µM on SHSY5Y.				[4,5]
**[D-Val⁹]laxaphycin A**	IC50 5.6 µM on SHSY5Y.				[5]
**[des-Gly¹¹]acyclolaxaphycin A**	10 µM, 50% deaths of SHSY5Y.				[5]
**[des-(Leu¹⁰-Gly¹¹)]acyclolaxaphycin A**	NA on SHSY5Y.				[5]
**Hormothamnin A**	IC50 0.2 µg/mL on SW-1271. IC50 0.16 µg/mL on A529. IC50 0.13 µg/mL on B16-F10. IC50 0.72 µg/mL on HCT116.	NA on *S. aureus*, *S. faecalis*, *E. coli* and *S. typhimurium*. IC50 > 100 µg/mL on *B. subtilis.* IC50 > 100 µg/mL on *P. aeruginosa*.	NA on *C. albicans* and *T. mentagrophytes.*	LD50 ~ 5 µg/mL on goldfish *C. carassius*. 20 µg/mL, NA on brine shrimp.	[9]
**Lobocyclamide A**			MIC 100 µg/disc on *C. albicans* (inhibition zone of 7 mm for 150 µg/disc). Synergy with lobocyclamide B (MIC 10-30 µg/mL, 1:1).		[10]
**Scytocyclamide A**	10 µg/mL, active on CaCo 2, HeLa, Swiss 3T3, Ebl, Rbl, LoVo and 293 cell lines.	64 µg/mL, NA on *S. aureus, S. pyogenes, E. coli, B. subtilis, E. faecium, A. baumannii, C. pseudodiphtericum.*	64 µg/mL, NA on *C. albicans.* Active on *A. flavus* (inhibition zone of 10 mm for 200 µg). Synergy with scytocyclamide B (inhibition zone of 36 mm for 100 µg A + 300 µg B) and with scytocyclamide C (inhibition zone of 33 mm for 100 µg A + 80 µg C).	NA on *D. magna*. LD50 12 µmol on *T. platyurus*.	[11,12]
**Scytocyclamide A2**			Active on *A. flavus* (inhibition zone of 7 mm for 200 µg). Synergy with scytocyclamide B2 (inhibition zone of 24 mm for 100 µg A2 + 43 µg B2) and with scytocyclamide B3 (inhibition zone of 25 mm for 100 µg A2 + 43 µg B3).		[12]

**Trichormamide A**	IC50 9.9 μM on MDA-MB-435 cells. IC50 16.9 μM on HT-29 cells.			[13]
**Trichormamide D**	IC50 11.7 μM on MDA-MB-435 cells. IC50 11.5 μM on HT-29 cells.	50 μg/mL, NA on *M. tuberculosis, M. smegmatis, S. aureus* and *E. coli.*	50 μg/mL, NA on *C. albicans*.	[14]
**Heinamides A1-A3**		NA on *S. aureus, E. faecium, B. cereus, M. luteus, P. aeruginosa, E. coli, A. baumannii, E. aerogenes, S. enterica.*	Crude extract of heinamides A and B inhibits *A. flavus*. NA on *C. albicans, C. guillermondi, C. krusei, C. parapsilosis, F. neoformans, A. niger, A. parasiticus.*	[15]

Table S2. Summary of the biological activity of laxaphycins B and their derivatives. For the purpose of clarity, the intracellular properties of the peptides have not been reported. Dose, Activity, Target. NA=Not active.

Laxaphycin B family (12 residues)	Cytotoxicity	Antimicrobial activity	Antifungal activity	Toxicity on animals	Ref.
**Laxaphycin B**	MIC 0.12 µg/mL on KB cells. Synergy with laxaphycin A (MIC 0.10 µg/mL with 0.06 µg A+ 0.05 µg B). IC50 1.11 µM on CCRF-CEM-WT cells. IC50 1.02 µM on CEM/VLB cells. IC50 1.37 µM on CEM/VM1 cells. Synergy with laxaphycin A (at 1 µM) IC50/3. IC50 0.5 ± 0.07 µM with Ho and 1 ± 0.12 µM with RRT on MCF7. IC50 0.19 ± 0.03 µM on PA1. IC50 0.58 ± 0.03 µM with Ho and 0.8 ± 0.1 µM with RRT on PC3. IC50 between 3 and 6 µM on DLD1. IC50 0.3 ± 0.03 µM with Ho and 0.4 ± 0.03 µM with RRT on M4Beu. IC50 < 2 µM on A549. IC50 < 2 µM on human normal fibroblasts and L-929 murine immortalized cells. IC50 0.3 µM with MTT and 1.8 µM with LDH on SHSY5Y.	NA on *E. coli* and *S. enterica* Typhimurium. MIC 250 µg/mL on *S. aureus*, *B. cereus*, *L. monocytogenes*.	MIC 64 µg/mL on *A. oryzae*. Moderate activity on *C. albicans*, *S. cerevisiae* and *T. mentagrophytes*. Synergism with laxaphycin A (MIC 16 µg/mL with 9.6 µg A+ 6.4 µg B).	200 µg/mL, lethal on *A. salina.*	[1–3,6–8,16]
**Laxaphycin B2**	IC50 4.15 ± 0.31 µM on CCRF-CEM-WT cells. IC50 3.33 ± 0.08 µM on CEM/VLB cells. IC50 4.14 ± 0.09 µM on CEM/VM1 cells.				[3]
**Laxaphycin B3**	IC50 1.50 ± 0.06 µM on CCRF-CEM-WT cells. IC50 1.35 ± 0.05 µM on CEM/VLB cells. IC50 1.45 ± 0.11 µM on CEM/VM1 cells. IC50 0.15 µM with MTT and 0.8 µM with LDH on SHSY5Y.	NA on *E. coli* and *S. enterica* Typhimurium. MIC 500 µg/mL on *S. aureus*. MIC 250 µg/mL on *B. cereus*. NA on *L. monocytogenes*.			[3,8,16]
**Laxaphycin B4**	IC50 1.7 µM on HCT116 cells.				[4]
**Laxaphycin B5**	IC50 1.2 µM on MDA-MB-435. IC50 2.2 µM on MDA-MB-231. IC50 1.6 µM on OVCAR3.				[17]
**Laxaphycin B6**	IC50 0.58 µM on MDA-MB-435. IC50 0.81 µM on MDA-MB-231. IC50 0.92 µM on OVCAR3.				[17]
**Laxaphycin D = Scytocyclamide B**	10 µg/mL, active on CaCo 2, HeLa, Swiss 3T3, Ebl, Rbl, LoVo and 293 cell lines.	64 µg/mL, NA on *S. aureus*, *S. pyogenes*, *E. coli*, *B. subtilis*, *E.*	64 µg/mL, NA on *C. albicans.* Inhibits *A. flavus* (inhibition zone of 23 mm for 600 µg). Synergy with scytocyclamide A (inhibition	NA on *D. magna*.	[11,12]

		faecium, A. baumannii, C. pseudodiphtericum	zone of 36 mm for 100 μg A + 300 μg B) and with scytocyclamide C (inhibition zone of 23 mm for 300 μg B + 80 μg C).	LD50 4 μmol on *T. platyurus*.	
**Acyclolaxaphycin B**	Until 10 μM, NA on SHSY5Y cells.				[16]
**Acyclolaxaphycin B3**	Until 10 μM, NA on SHSY5Y cells.				[16]
**[des-(Ala^{4}-Hle^{5})]acyclolaxaphycin B**	NA on SHSY5Y cells.				[16]
**[des-(Ala^{4}-Hle^{5})]acyclolaxaphycin B3**	NA on SHSY5Y cells.				[16]
**Lobocyclamide B**			MIC 30-100 μg/mL on *C. albicans* (inhibition zone of 8 mm for 150 μg/disc). Synergy with lobocyclamide A (MIC 10-30 μg/mL, 1:1). Activity on *C. glabrata* (inhibition zone of 6 mm for 150 μg/disc).		[10]
**Lobocyclamide C**			Activity on *C. albicans* (inhibition zone of 10 mm for 150 μg/disc). Activity on *C. glabrata* (inhibition zone of 8 mm for 150 μg/disc).		[10]
**Scytocyclamide B2**			Inhibits *A. flavus* (inhibition zone of 10 mm for 85 μg). Synergy with scytocyclamide A2 (inhibition zone of 24 mm for 100 μg A2 + 43 μg B2).		[12]
**Scytocyclamide B3**			Inhibits *A. flavus* (inhibition zone of 20 mm for 85 μg). Synergy with scytocyclamide A2 (inhibition zone of 25 mm for 100 μg A2 + 43 μg B3).		[12]
**Scytocyclamide C**		64 μg/mL, NA on *S. aureus*, *S. pyogenes, E. coli, B. subtilis, E. faecium, A. baumannii, C. pseudodiphtericum*	64 μg/mL, NA on *C. albicans.* Inhibits *A. flavus* (inhibition zone of 22 mm for 160 μg). Synergy with scytocyclamide A (inhibition zone of 33 mm for 100 μg A + 80 μg C) and with scytocyclamide B (inhibition zone of 23 mm for 300 μg B + 80 μg C).		[11,12]
**Lyngbyacyclamide A**	IC50 0.7 μM on B16 mouse melanoma cells.			70 μM, NA on brine shrimp (*Artemia* genus)	[18]
**Lyngbyacyclamide B**	IC50 0.7 μM on B16 mouse melanoma cells.			70 μM, NA on brine shrimp (*Artemia* genus)	[18]
**Trichormamide B**	IC50 0.8 μM on MDA-MB-435 cells. IC50 1.5 μM on HT-29 cells.				[13]

**Trichormamide C**	IC50 1 μM on MDA-MB-435 cells. IC50 1.7 μM on HT-29 cells.	MIC 23.8 μg/mL on *M. tuberculosis.* 50 μg/mL, NA on *M. smegmatis, S. aureus* and *E. coli.*	50 μg/mL, NA on *C. albicans*.	[14]
**Heinamides B1-B5**		NA on *S. aureus, E. faecium, B. cereus, M. luteus, P. aeruginosa, E. coli, A. baumannii, E. aerogenes, S. enterica.*	Crude extract of heinamides A and B inhibits *A. flavus*. NA on *C. albicans, C. guillermondi, C. krusei, C. parapsilosis, F. neoformans, A. niger, A. parasiticus.*	[15]

References

1. Frankmolle, W.P.; Larsen, L.K.; Caplan, F.R.; Patterson, G.M.L.; Knubel, G.; Levine, I.A.; Moore, R.E. Antifungal Cyclic Peptides from the Terrestrial Blue-Green Alga Anabaena Laxa. I. Isolation and Biological Properties. *J. Antibiot. (Tokyo)* **1992**, *45*, 1451–1457, doi:10.7164/antibiotics.45.1451.
2. Bonnard, I.; Rolland, M.; Francisco, C.; Banaigs, B. Total Structure and Biological Properties of Laxaphycins A and B, Cyclic Lipopeptides from the Marine CyanobacteriumLyngbya Majuscula. *Lett. Pept. Sci.* **1997**, *4*, 289–292, doi:10.1007/BF02442891.
3. Bonnard, I.; Rolland, M.; Salmon, J.-M.; Debiton, E.; Barthomeuf, C.; Banaigs, B. Total Structure and Inhibition of Tumor Cell Proliferation of Laxaphycins. *J. Med. Chem.* **2007**, *50*, 1266–1279, doi:10.1021/jm061307x.
4. Cai, W.; Matthew, S.; Chen, Q.-Y.; Paul, V.J.; Luesch, H. Discovery of New A- and B-Type Laxaphycins with Synergistic Anticancer Activity. *Bioorg. Med. Chem.* **2018**, *26*, 2310–2319, doi:10.1016/j.bmc.2018.03.022.
5. Bornancin, L.; Alonso, E.; Alvariño, R.; Inguimbert, N.; Bonnard, I.; Botana, L.M.; Banaigs, B. Structure and Biological Evaluation of New Cyclic and Acyclic Laxaphycin-A Type Peptides. *Bioorg. Med. Chem.* **2019**, *27*, 1966–1980, doi:10.1016/j.bmc.2019.03.046.
6. Bonnard, I.; Bornancin, L.; Dalle, K.; Chinain, M.; Zubia, M.; Banaigs, B.; Roué, M. Assessment of the Chemical Diversity and Potential Toxicity of Benthic Cyanobacterial Blooms in the Lagoon of Moorea Island (French Polynesia). *J. Mar. Sci. Eng.* **2020**, *8*, 406, doi:10.3390/jmse8060406.
7. Gbankoto, A.; Vigo, J.; Dramane, K.; Banaigs, B.; Aina, E.; Salmon, J.-M. Cytotoxic Effect of Laxaphycins A and B on Human Lymphoblastic Cells (CCRF-CEM) Using Digitised Videomicrofluorometry. *In Vivo* **2005**, *19*, 577–582.
8. Dussault, D.; Vu, K.D.; Vansach, T.; Horgen, F.D.; Lacroix, M. Antimicrobial Effects of Marine Algal Extracts and Cyanobacterial Pure Compounds against Five Foodborne Pathogens. *Food Chem.* **2016**, *199*, 114–118, doi:10.1016/j.foodchem.2015.11.119.
9. Gerwick, W.H.; Mrozek, Ch.; Moghaddam, M.F.; Agarwal, S.K. Novel Cytotoxic Peptides from the Tropical Marine Cyanobacterium Hormothamnion Enteromorphoides 1. Discovery, Isolation and Initial Chemical and Biological Characterization of the Hormothamnins from Wild and Cultured Material. *Experientia* **1989**, *45*, 115–121, doi:10.1007/BF01954842.
10. MacMillan, J.B.; Ernst-Russell, M.A.; de Ropp, J.S.; Molinski, T.F. Lobocyclamides A−C, Lipopeptides from a Cryptic Cyanobacterial Mat Containing *Lyngbya Confervoides*. *J. Org. Chem.* **2002**, *67*, 8210–8215, doi:10.1021/jo0261909.
11. Grewe, J.C. Cyanopeptoline und Scytocyclamide: Zyklische Peptide aus *Scytonema hofmanni* PCC 7110 Struktur und biologische Aktivität. Doctoral dissertation, Universität Freiburg im Breisgau, 2005.
12. Heinilä, L.M.P.; Fewer, D.P.; Jokela, J.K.; Wahlsten, M.; Jortikka, A.; Sivonen, K. Shared PKS Module in Biosynthesis of Synergistic Laxaphycins. *Front. Microbiol.* **2020**, *11*, doi:10.3389/fmicb.2020.578878.
13. Luo, S.; Krunic, A.; Kang, H.-S.; Chen, W.-L.; Woodard, J.L.; Fuchs, J.R.; Swanson, S.M.; Orjala, J. Trichormamides A and B with Antiproliferative Activity from the Cultured Freshwater Cyanobacterium *Trichormus* Sp. UIC 10339. *J. Nat. Prod.* **2014**, *77*, 1871–1880, doi:10.1021/np5003548.
14. Luo, S.; Kang, H.-S.; Krunic, A.; Chen, W.-L.; Yang, J.; Woodard, J.L.; Fuchs, J.R.; Hyun Cho, S.; Franzblau, S.G.; Swanson, S.M.; et al. Trichormamides C and D, Antiproliferative Cyclic Lipopeptides from the Cultured Freshwater Cyanobacterium Cf. Oscillatoria Sp. UIC 10045. *Bioorg. Med. Chem.* **2015**, *23*, 3153–3162, doi:10.1016/j.bmc.2015.04.073.
15. Heinilä, L.M.P.; Fewer, D.P.; Jokela, J.K.; Wahlsten, M.; Ouyang, X.; Permi, P.; Jortikka, A.; Sivonen, K. The Structure and Biosynthesis of Heinamides A1–A3 and B1–B5, Antifungal Members of the Laxaphycin Lipopeptide Family. *Org. Biomol. Chem.* **2021**, *19*, 5577–5588, doi:10.1039/D1OB00772F.
16. Alvariño, R.; Alonso, E.; Bornancin, L.; Bonnard, I.; Inguimbert, N.; Banaigs, B.; Botana, L.M. Biological Activities of Cyclic and Acyclic B-Type Laxaphycins in SH-SY5Y Human Neuroblastoma Cells. *Mar. Drugs* **2020**, *18*, 364, doi:10.3390/md18070364.
17. Sullivan, P.; Krunic, A.; Burdette, J.E.; Orjala, J. Laxaphycins B5 and B6 from the Cultured Cyanobacterium UIC 10484. *J. Antibiot. (Tokyo)* **2020**, *73*, 526–533, doi:10.1038/s41429-020-0301-x.
18. Maru, N.; Ohno, O.; Uemura, D. Lyngbyacyclamides A and B, Novel Cytotoxic Peptides from Marine Cyanobacteria Lyngbya Sp. *Tetrahedron Lett.* **2010**, *51*, 6384–6387, doi:10.1016/j.tetlet.2010.06.105.
